# Single-cell RNA sequencing reveals the contribution of smooth muscle cells and endothelial cells to fibrosis in human atrial tissue with atrial fibrillation

**DOI:** 10.1186/s10020-024-00999-1

**Published:** 2024-12-19

**Authors:** Na An, Fan Yang, Guoxia Zhang, Yuchen Jiang, Haoqi Liu, Yonghong Gao, Yingjian Li, Peifeng Ji, Hongcai Shang, Yanwei Xing

**Affiliations:** 1 Guang’anmen Hospital, Chinese Academy of Chinese Medical Sciences, No.5 Beixian’ge Street, Xicheng District, Beijing, 100053 China; 2 Key Laboratory of Chinese Internal Medicine of Ministry of Education, Dongzhimen Hospital, Beijing University of Chinese Medicine, No.5 Haiyuncang, Dongcheng District, Beijing, 100700 China; 3 Beijing University of Chinese Medicine, Beijing, 100029 China; 4 Beijing Anzhen Hospital, Capital Medical University, Beijing, 100029 China; 5 Computational Genomics Lab, Beijing Institutes of Life Science, Chinese Academy of Sciences, No. 5, Yard 1, Beichen West Road, Chaoyang District, Beijing, 100101 China

**Keywords:** Atrial fibrillation, Cardiovascular disease, Single-cell sequencing, Genetics, Genome-wide association study

## Abstract

**Aims:**

Atrial fibrillation (AF) has high mortality and morbidity rates. However, the intracellular molecular complexity of the atrial tissue of patients with AF has not been adequately assessed.

**Methods and results:**

We investigated the cellular heterogeneity of human atrial tissue and changes in differentially expressed genes between cells using single-cell RNA sequencing, fluorescence in situ hybridization, intercellular communication, and cell trajectory analysis. Using genome-wide association studies (GWAS) and proteomics, we discovered cell types enriched for AF susceptibility genes. We discovered eight different cell types, which were further subdivided into 23 subpopulations. In AF, the communication strength between smooth muscle cells (SMCs) and fibroblast (FB) 3 cells increased and the relevant signaling pathways were quite similar. Subpopulations of endothelial cells (ECs) are mainly involved in fibrosis through *TXNDC5* and *POSTN*. AF susceptibility genes revealed by GWAS were especially enriched in neuronal and epicardial cells, FB3, and lymphoid (Lys) cells, whereas proteomic sequencing differential proteins were concentrated in FB3 cells and SMCs.

**Conclusions:**

This study provides a cellular landscape based on the atrial tissue of patients with AF and highlights intercellular changes and differentially expressed genes that occur during the disease process. A thorough description of the cellular populations involved in AF will facilitate the identification of new cell-based interventional targets with direct functional significance for the treatment of human disease.

**Supplementary Information:**

The online version contains supplementary material available at 10.1186/s10020-024-00999-1.

## Introduction

Atrial fibrillation (AF) has considerably high mortality and morbidity rates (Chugh et al. 2014), and current pharmacological strategies for the treatment of AF have limited efficacy and possible undesirable effects (Heijman et al. 2018). In a Cochrane collaborative analysis of randomized blinded trials of antiarrhythmic drugs to maintain sinus rhythm (SR) after cardioversion, nearly all antiarrhythmic drugs had significant arrhythmic potential and high mortality (Lafuente-Lafuente et al. 2015; Valembois et al. 2019). Moreover, the catheter ablation of AF features major complications, including cardiac tamponade and stroke/transient ischemic attack (Pothineni et al. 2014). The adverse effects of AF therapy have stimulated the exploration of new knowledge of the human heart. An increasing number of cardiac physiology and pathology mechanisms have identified cellular heterogeneity as a major focus (Churko et al. 2018).

Atrial fibrosis is a major pathophysiological factor for initiation and maintenance of AF (Sohns and Marrouche 2020). Henderson et al. discussed that increasingly sophisticated experimental techniques have revealed substantial diversity and functional heterogeneity within fibroblast populations during organ fibrosis (Henderson et al. 2020). There are also studies that utilize single-cell RNA sequencing (scRNA-seq) to investigate fibrosis mechanisms in mouse hearts (Soliman et al. 2020; Dick et al. 2019). However, the influence of various cell types on fibrosis formation in the pathological process of patients with AF remains unclear.

The scRNA-seq has enabled exploration of the cellular heterogeneity of the human atrium in SR and AF. For example, heart-resident macrophages directly modulated the electrical properties of cardiomyocytes in a recent report (Hulsmans et al. 2017). Consequently, explaining the principles that underlie cellular composition and cell–cell communication is a crucial step in increasing our understanding that will greatly facilitate the treatment of AF. However, efforts in this area are lacking, mainly due to difficulty obtaining human heart tissue samples and technical limitations.

Additionally, genome-wide association studies (GWAS) and proteomics have identified many loci (Roselli et al. 2018, Weng et al. 2017; Sakaue et al. 2021; Nielsen et al. 2018; Nielsen et al. 2018; Low et al. 2017; Lee et al. 2017; Larson et al. 2007; Kertai et al. 2015; Jiang et al. 2021; Hong et al. 2021; He et al. 2016; Gudbjartsson et al. 2009; Gudbjartsson et al. 2007; Ellinor et al. 2010; Ellinor et al. 2012; Christophersen et al. 2017; Benjamin et al. 2009) and proteins associated with an increased risk of AF. However, the translation of these findings into new therapeutic approaches is hampered by the lack of information on cell-specific expression patterns of candidate drug genes for specific AF cell communities and disease loci. Therefore, using single-cell sequencing data to identify AF-related genes and proteins that are highly expressed in particular cell types will efficiently identify specific candidates for functional follow-up.

Therefore, we performed scRNA-seq on samples of the right atrial appendages of patients obtained during coronary bypass grafting and/or valve replacement and we aimed to elucidate the cellular composition and communication of atrial tissue in patients with AF to provide strategic implications for its prevention and intervention.

## Research design and methods

### Data availability

Detailed methods are available in Supplementary Material. The data, methods, and study materials will be made available to other researchers upon request.

### Sample preparation from human participants

Right atrial appendages of patients (*n* = 18) undergoing coronary bypass grafting and/or valve replacement were collected. Proteomic technology samples were flash-frozen and refrigerated at -80 °C. Freshly acquired samples were used to isolate single cells. The tissue was formalin-fixed paraffin-embedded for mRNA Fluorescence in Situ Hybridization (mFISH) analysis.

### Single-cell preparation and single-cell RNA sequencing

Each patient’s right atrial appendage was collected and re-suspended at a concentration of 1 × 10^6^/mL in Dulbecco’s phosphate-buffered saline containing 0.04% bovine serum albumin. Employing the Miltenyi Tissue Dissociation Kits, in accordance with the manual, the tissue samples were digested. Briefly, the enzyme solutions (Enzyme D, Enzyme R, and Enzyme A) were configured initially. 3 mL of serum-free DMEM medium was added to the dry powder of Enzyme D, and left for 5 min with inversion and mixing at 1 min intervals. 2.7 mL of serum-free DMEM medium was added to the dry powder of Enzyme R. 1 mL of Buffer A was added to the dry powder of Enzyme A. Mix 2.35 mL of serum-free DMEM, 100 µL of Enzyme D solution, 50 µL of Enzyme R solution, and 12.5 µL of Enzyme A solution uniformly. Subsequently, the tissue digestion was carried out. Run the Multi_H program. After centrifugation, the suspended cells were placed into a 100 µm SmartStrainer filter, and the supernatant was discarded after filtration. Resuspend the cells with an appropriate amount of buffer, and the cell suspension is used for subsequent single-cell experiments. To eliminate cell aggregates, cells with a greater aggregation rate were filtered out (as determined by a Countstar cell count and analysis system). The cell suspensions with > 90% live cells assessed by the Countstar were placed onto a Chromium Single Cell Controller (10× Genomics). Single-cell RNA-seq libraries were prepared using a Single Cell 3’ Library Gel Bead Kit V2 following the manufacturer’s instructions. On an Illumina Hiseq X-ten platform, the scRNA-seq libraries were sequenced using 150-bp paired-end reads.

### Statistical analysis

Data are expressed as the mean ± SD. Two-sided unpaired Student’s t-tests was used to compare two groups with a normal distribution. Statistical analyses were performed using GraphPad Prism (version 9.0, GraphPad Software Incorporate, La Jolla, CA, USA). Statistical significance is indicated by asterisks; ^*^P < 0.05 and ^**^P < 0.01. The number of biological replicates for each experiment is provided individually in the corresponding figure legends.

## Results

### Use of scRNA-seq revealed the cellular composition of the human atrium in SR or AF

**Fig. 1:**
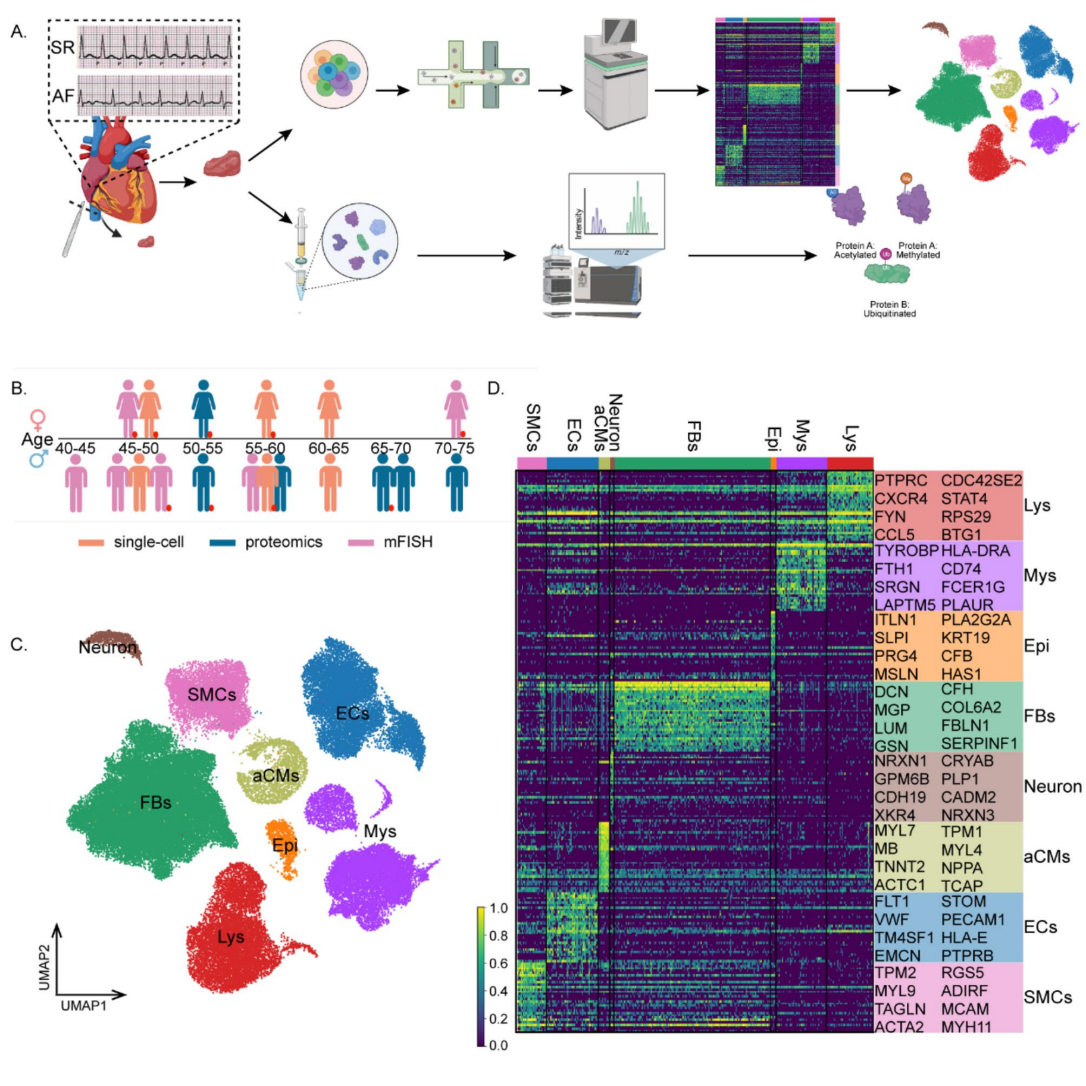
Overview of the cell composition of the adult human atrial in SR and AF. **A**. Illustration of workflow of scRNA-seq and proteomics in human SR and AF atrial samples. **B**. Infographic shows donors (women, top; men, bottom), age, and group (red circle, AF) (*n* = 18, scRNA-seq to the right atrial appendages of 6 patients. Samples were collected from another 12 patients for proteomics and FISH analyses). Data are available in Table S1. **C**. Uniform manifold approximation and projection (UMAP) embedding of 71,440 cells reveals 8 cellular clusters. Clusters are distinguished by different colors. **D**. Heatmap of differentially expressed genes. For each cluster, the genes and their relative expression levels in all sequenced cells are shown. Selected genes for each cluster are color-coded and shown on the right

To comprehensively delineate the effect of AF on different cell types, we applied scRNA-seq to the right atrial appendages of patients (50% female). Samples were collected from another patients for proteomics and FISH analyses (16.7% female in the proteomics analysis; 33% female in the FISH analysis) (Fig. 1a and b). After ensuring stringent quality control, we applied unbiased clustering on 71,440 cells (SR: 37,450; AF: 33,990). Based on Unsupervised Uniform Manifold Approximation and Projection (UMAP) and established cell type-specific marker genes, we first assigned these clusters into eight major cell types. Other cell types were then isolated and further clustered into 23 cell subclusters using lineage-specific marker genes (Figs. S1a, S1c). Cells of the FB compartment highly express *DCN* and *GSN* in six subclusters. The SMC lineage was identified by *MYH11* and *TAGLN*, while the atrial cardiomyocytes (aCMs) lineage was identified by *MYL7* and *NPPA*. We identified three major ECs subclusters that express canonical markers such as *VWF* and *PECAM1* and observed two immune cell clusters, including myeloid cells (Mys) (expressing *TYROBP*, *CD74*, and *HLA-DRA*) and lymphoid cells (Lys) (expressing *PTPRC* and *CXCR4*) (Fig. 1c and d; Table S2).

Atrial tissues contained 44% FBs, 27.3% immune cells (myeloid and lymphoid), 14.6% ECs, 8.1% SMCs, 3.2% aCMs, 1.5% epicardial cells, and 1.2% neuronal cells (Figs. S1d, S1e). The number of genes and UMI detected per cell are shown in Figs. S1f and S1g.

### Characterization of cardiac fibroblasts at single-cell resolution

We analyzed the changes in the proportion of cell clusters in the SR and AF. For the study, by comparing UMAP and cell ratios, we observed a reduction in the ratio of cell types or subtypes such as aCMs, ECs, and epicardial cells in the AF and an increase in the proportion of FBs and Mys (Figs. S1b, S2a). However, due to the limitation of sample size, further researches may be needed to verify it in the future.

**Fig. 2:**
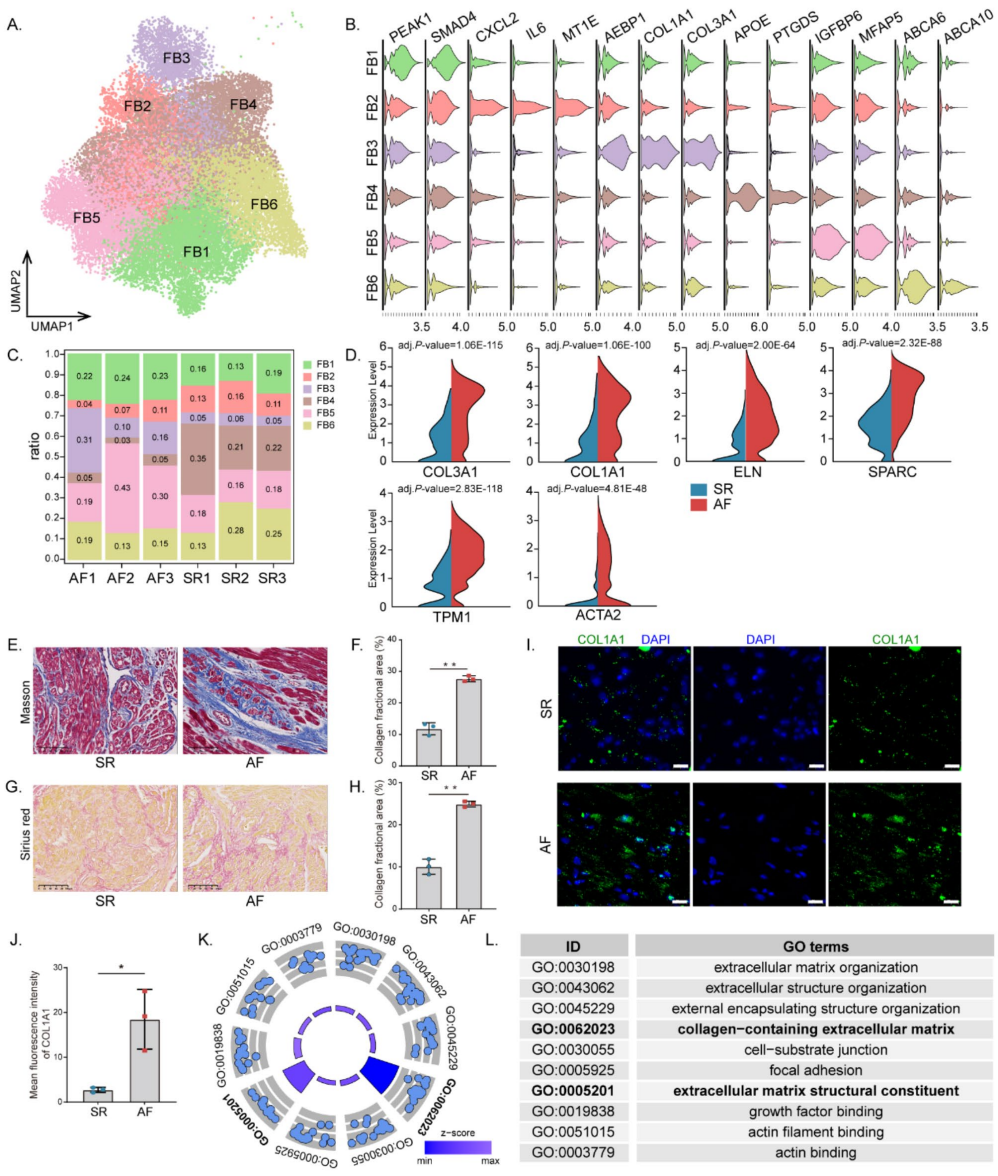
Fibroblasts subcluster into distinct cell populations. **(A)** UMAP embedding of 6 distinct fibroblasts cell populations. **(B)** Violin plots of fibroblasts cell-specific markers. **(C)** Cell proportions of fibroblast subclusters in SR and AF. Cells of FB3 were significantly increased in AF samples compared to SR samples. **(D)** Violin plots showing representative differentially expressed genes between SR (*n* = 3) FB3 and AF (*n* = 3) FB3. **(E)** Masson’s trichrome staining, Scale bar = 100 μm. **(F)** quantification of atrial fibrosis (blue) in SR (*n* = 3) and AF (*n* = 3). **(G)** Sirius red staining, Scale bar = 100 μm. **(H)** quantification of type I collagen deposition (red) in SR (*n* = 3) and AF (*n* = 3). **I**,**J.** Fluorescence in Situ Hybridization and quantification of COL1A1 in SR (*n* = 3) and AF (*n* = 3) (The images from at least five randomly chosen fields from per sample). Scale bar = 20 μm. **K**,**L.** GO Biological Process enrichment analysis of differentially expressed genes in FB3 between SR and AF. Blue circles represent up-regulated genes in AF. Data are expressed as the mean ± SD. Two-sided unpaired Student’s t-tests was used to compare two groups with a normal distribution. Statistical significance is indicated by asterisks; ^*^P < 0.05 and ^**^P < 0.01

We performed unsupervised clustering of all SR and AF fibroblasts and further analyzed the heterogeneity of the six subclusters, FB1–FB6 (Fig. 2a). These cells revealed common specific markers of fibroblast lineage and were enriched in known fibroblast genes such as *DCN* and *GSN*. We could assign FB phenotypes to the subclusters by evaluating marker genes (Fig. 2b). FB1 cells showed distinct expression of *PEAK1* and SMAD4. The FB2 cluster displayed enriched expression of the inflammation-associated genes *CXCL* and *IL6*. Importantly, subcluster FB3, which was significantly increased in AF, was enriched for *AEBP1*, a positive regulator of collagen fibrillogenesis via *COL1A1* and *COL3A1*. FB4 cells express genes responsive to lipids, for example, *APOE* and *PTGDS*. FB5 cells highly express *IGFBP6* and *MFAP5*, which are associated with growth factors. In addition, FB6 cells preferentially express *ABCA6* and − 10, members of the ATP binding cassette subfamily of transmembrane transporters. Additionally, PCOLCE2 was increased in FB1 and FB5, while RARRES2 was increased in FB2 and FB4 (Fig. S2c).

Next, SR and AF were compared in terms of the proportion of each fibroblast subpopulation. Comparatively to SR, AF tissues had increased proportions of FB1, FB3, and FB5 (FB3 in particular) and decreased proportions of FB2, FB4, and FB6 (Fig. 2c, S2b).

To further evaluate the pathologic progress of AF, we compared differences between AF fibroblasts and SR fibroblasts, especially FB3 cells. We identified that collagen and elastin, ECM, and actin differentiation-associated genes, such as *COL3A1*, *ELN*, *SPARC*, *TPM1*, and *ACTA2*, were significantly elevated in FB3 of AF (Fig. 2d, S2d, Table S3). To elucidate the structural remodeling changes in AF, we evaluated the changes in human atrial (right atrial appendages) pathology. The results of HE staining showed that the atrial muscle tissue of AF group was disordered and the myofilaments gap was increased, while the myocardium of SR group was orderly and compact (Fig. S2e). The results of masson’s trichrome staining and sinus red staining indicated that compared to the SR group, the AF showed myocardial fibrosis and collagen deposition, and that the fibrotic area of atrial in the AF group was significantly greater than that of SR (Fig. 2e and h). The mFISH results showed that the expression of COL1A1 was higher in AF than in SR (Fig. 2i and j). Gene Ontology (GO) analyses indicated that upregulated genes in the FB3 cell subcluster of AF were related to collagen-containing ECM, ECM structural constituent, and actin filament binding (Fig. 2k and l), whereas downregulated genes including *MT2A* and *MT1X* were associated with response to metal ions (Figs. S2f, S2g).

### Functional diversity of SMCs and ECs subtypes

**Fig. 3:**
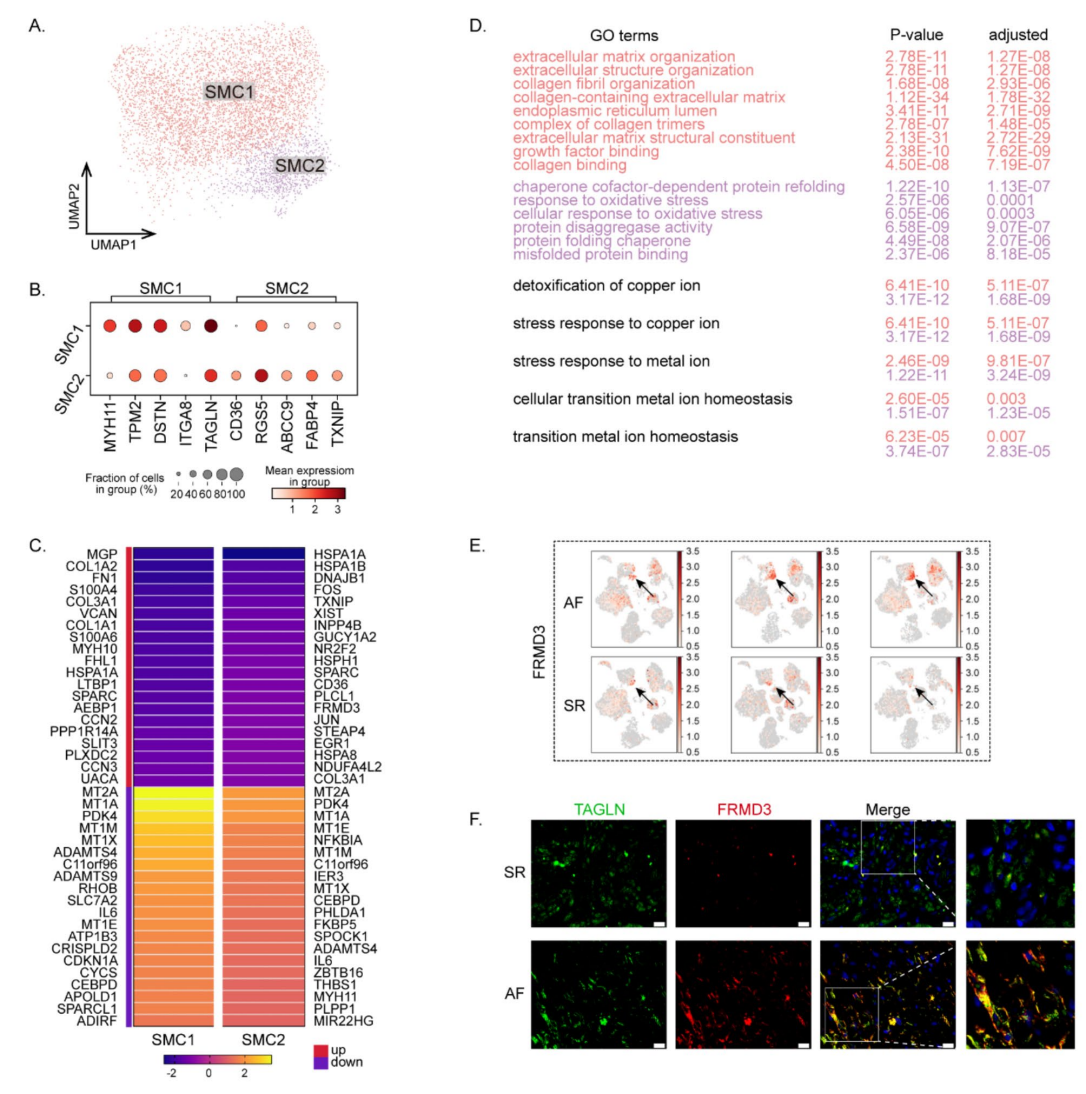
Subclustering of smooth muscle cells. **(A)** UMAP plot representing the 2 observed SMC subcluster. **(B)** Dot plot detailing the percentage of cells where each gene is detected (dot size) and mean expression (red) for representative subcluster marker genes. **(C)** Differentially expressed genes of up and down regulators of the SMC subsets. **(D)** GO enrichment terms of differentially expressed genes in SMCs. **(E)** Feature plots of the expression distribution for FRMD3 in AF and SR. Expression levels for each cell are color-coded and overlaid onto the UMAP plot. **(F)** Fluorescence in Situ Hybridization of TAGLN and FRMD3 in SR and AF. Scale bar = 20 μm

SMCs that express MYL9 and ACTA2 were split into two populations (Figs. 1d and 3a). Fig. S3a shows the cell proportions of the SMC subclusters for AF versus SR. SMC1 cells expressed transcripts indicating that muscle contraction (Fig. 3b), including *MYH11*, is the most specific for the smooth muscle lineage and the most definitive marker of SMC differentiation. SMC2 cells expressed fairly higher levels of inflammatory-related *CD36*, *TXNIP*, and *FABP4*, an adipocyte molecule. They also expressed subunits of ATP-sensitive potassium channels, *ABCC9*, and proliferation-associated *RGS5* (Fig. 3b). Despite the low expression of *MYH11*, SMC2 cells highly expressed the SMC-specific marker *TAGLN*, thus, we classified it as an SMC subcluster.

We observed the top 20 differentially expressed genes (DEGs) between the two subclusters (Fig. 3c). The upregulated genes of SMC1 exhibited functional enrichment in GO relevant to ECM organization, extracellular structure organization, and collagen fibril organization (Fig. 3d). However, SMC2 were mainly involved in processes that are related to chaperone cofactor-dependent protein refolding, responses to oxidative stress, and protein disaggregate activity. Except for genes related to ECM and fibrosis, we found ion channel differentiation-associated genes such as *FHL1* and *PPP1R14A* in SMC1. Of note, *PPP1R14A* also increased in the FB3 cells of AF (Table S3). We also found significantly increased numbers of oncogenes, such as *FRMD3*, *GUCY1A2*, and *INPP4B*, among the DEGs of SMC2 (Fig. 3e-f, S3b). However, the role of oncogenes in the development of AF remains unclear.

**Fig. 4:**
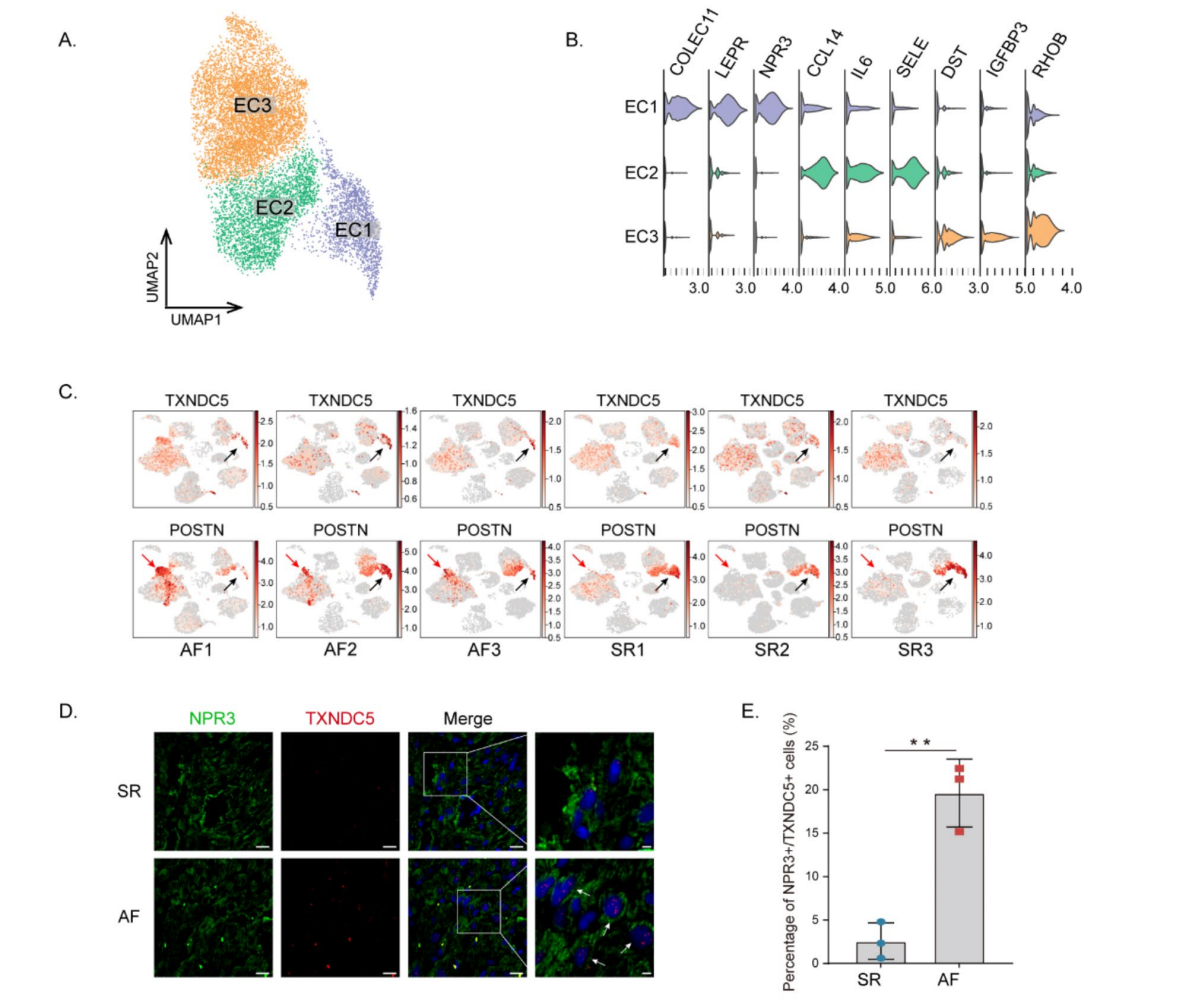
Atrial endothelial cells populations. **(A)** UMAP embedding of the ECs cell types. **(B)** Violin plots of ECs characterizing marker genes. **(C)** Feature plots of the expression distribution for TXNDC5 and POSTN in SR and AF. Expression levels for each cell are color-coded and overlaid onto the UMAP plot (black arrow, EC1; red arrow, FB3). **(D)** Fluorescence in Situ Hybridization of NPR3 and TXNDC5 in SR (*n* = 3) and AF (*n* = 3). Scale bar = 20 μm. Arrowheads indicate NPR3+/TXNDC5 + cells. Scale bar = 5 μm. **(E)** Percentage of NPR3+/TXNDC5 + cells in SR and AF. Data are presented as mean values ± SD (*n* = 3 images examined over 3 independent experiments). Statistical analysis was performed using unpaired two-tailed Student’s t-test, ^**^P = 0.0026

**Fig. 5:**
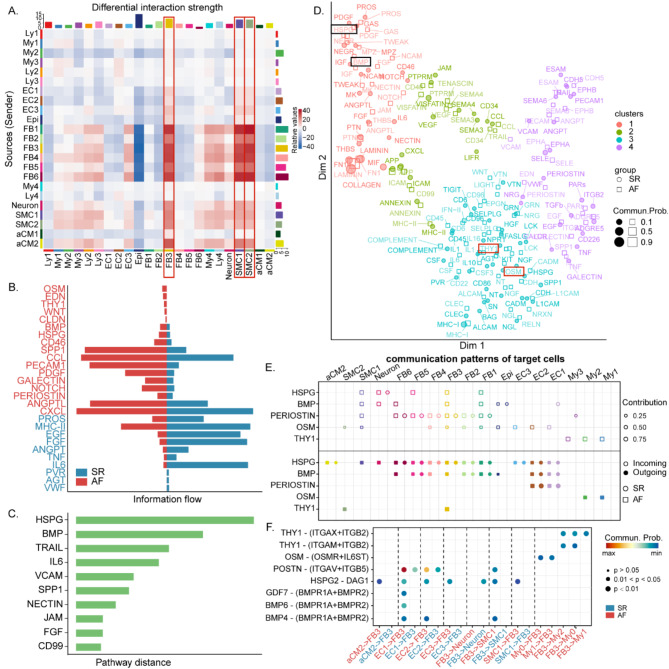
CellChat analysis of the communications between atrial cells of SR and AF. **(A)** The differential interaction strength of subclusters. **(B)** The significant signaling pathways were ranked based on their differences of overall information flow within the inferred networks between SR and AF. The overall information flow of a signaling network is calculated by summarizing all the communication probabilities in that network. The top signaling pathways colored by red are more enriched in AF, and the bottom ones colored by blue were more enriched in the SR. **(C)** The top 10 overlapping signaling pathways between SR and AF were ranked based on their pairwise Euclidean distance in the shared two-dimensional manifold. **(D)** Jointly projecting and clustering signaling pathways from SR and AF into a shared two-dimensional manifold according to their functional similarity. Circle and square symbols represent the signaling networks from SR and AF respectively. Each dot or square represents the communication network of one signaling pathway. **(E)** The dot plot showing the comparison of outgoing signaling patterns of secreting cells between SR and AF. The dot size is proportional to the contribution score computed from pattern recognition analysis. Higher contribution score implies the signaling pathway is more enriched in the corresponding cell group. **(F)** Comparison of the significant ligand-receptor pairs between SR and AF. Dot color reflects communication probabilities and dot size represents computed p-values. Empty space means the communication probability is zero. p-values are computed from one-sided permutation test

ECs identified by special markers *PECAM1* and *VWF* comprise three subclusters (Figs. 1d and 4a). A subset of cells in EC1 express *NPR3*, suggesting the EC1 population may represent endocardial cells. EC1 cells also significantly express *LEPR* and *COLLEC11*. EC2 cells express genes that correlate with antigen presentation and immune regulation (*CCL14*, *SELE*, and *IL6*), whereas EC3 cells express apoptosis and cell adhesion–related genes *DST*, *IGFBP3* and *RHOB* (Fig. 4b). Compared with SR, the proportions of the EC1 cell subclusters in AF are reduced (Fig. S3c). The upregulated genes in EC1 cells included ECM proteins, for instance, *BGN*, *CCN1*, *CCN2*, and *HSP1A1*. As such, EC1 cells displayed enriched migration and ECM (Figs. S3f, S3g). Significantly, a DEG analysis comparing SR and AF EC1 cells showed that specific DEGs were exhibited by EC1 cells, including *TXNDC5* and *POSTN* (Fig. 4c). The mFISH results demonstrated a higher proportion of NPR3+/TXNDC5 + cells in AF than in SR. Interestingly, TXNDC5 was mostly expressed in the nucleus of AF group (Fig. 4d and e). The secreted 90-kDa POSTN, a nonstructural component of the ECM, is a crucial stromal cytokine in cardiac mesenchymal tissue. Interestingly, DEG exploration revealed that POSTN was specifically upregulated in EC1 cells of SR versus both EC1 and FB3 cells of AF (Fig. 4c, S3d, S3e). POSTN plays a well-known role in fibrosis, but our study further demonstrated that, under normal conditions, POSTN exists in EC1 cells, and when stimulated by certain factors, communication between EC1 and FB3 cells may occur through it, leading to fibrosis. This result was also confirmed in cell communication analysis and PAGA analysis (Fig. 5f, S5c).

### Cell–cell communication analysis of SR versus AF

We investigated potential ligand–receptor interactions between cell types based on CellChat v2.0 to predict cell–cell communication under disease conditions. FB3 and SMCs displayed higher strengths of interactions (Fig. 5a). We also explored multifarious cell populations and signaling pathway coordination functions. This analysis revealed six patterns for outgoing and incoming signaling of SR and three patterns for outgoing and incoming signaling of AF. In the SR group, the communication patterns of target cells displaying that outcoming and incoming SMCs signaling focused on pattern #6, including signaling pathways VWF, PDGF, and AGT (Figs. S4a, S4b). It is noteworthy that, in AF, the incoming and outgoing signals of SMC shared pattern #1 with FBs. Pattern #1 involved a higher proportion of outgoing/incoming FBs and SMCs signaling in AF, representing numerous pathways including but not limited to PERIOSTIN, HSPG, BMP, SPP1, PROS, and THY1 (Figs. S4a, S4b). All incoming and outgoing EC signaling pathways are dominated by patterns #2 and #3, respectively, which also have higher portions in AF and represent such pathways as CXCL, PECAM1, NPR1, OSM, EDN, and IL6. We also investigated detailed changes in incoming/outgoing signaling of all crucial pathways in SR and AF utilizing pattern recognition analysis. We found that the signaling pathways of the SMCs and FBs were similar in AF, including COLLAGEN, LAMININ, and FN1, whereas there were significant differences among them in the SR group (Figs. S4c, S4d).

We next compared the information flow between the two groups and found that 19 of the 27 pathways were highly active in SR and AF. These possibly represent the core signaling pathways essential for human atrial function in the normal or disease state. Five pathways were active in AF only. These contain vital pathways for AF as OSM and THY1. Three signals are distinctively active in SR, including VWF, AGT, and IL6 (Fig. 5b). The Euclidean distance between any pair of the shared signaling pathways in the shared two-dimensional manifold was calculated; the top 10 are shown, and the HSPG and BMP pathways had large distances (Fig. 5c). We extrapolated cell–cell communication networks for the SR and AF, respectively, and then mapped them onto a shared two-dimensional manifold and divided them into groups according to their functional similarity. We defined four pathway groups (Fig. 5d). The HSPG and BMP signaling pathways were grouped together, as were COLLAGEN and LAMININ, indicating that these pathways have similar effects in the pathogenesis of AF. OSM and THY1, which are active only in AF, were in the same group as the immune-related pathways such as MHC-I, MHC-II, and IL-1. We used pattern recognition analysis to explore the specific changes in the incoming/outgoing signals of the above pathways (Fig. 5e). Two major results were obtained: (a) the HSPG and BMP signaling pathways were driven by ECs and FBs and PERIOSTIN was dominantly driven by ECs; (b) in AF, Mys cells were the origin of OSM ligands and FB3 was the predominant source of THY1 ligands, with partial contributions from SMC2. Furthermore, we analyzed the cell crosstalk by HSPG and BMP signaling pathways. As shown in Figs. S6e, S6f, compared with the SR group, the AF group demonstrated extensive intercellular communication between ECs, especially EC1 and other cell types, including FBs and SMC1. Specific to PERIOSTIN signaling, CellChat distinguished ligand–receptor pair POSTN-(ITGAV + ITGB5) as the most prominent, which serves as the primary signaling from EC1 and EC2 to FB3 in the AF versus SR (Fig. 5f). This result is similar to previously mentioned results, which found that POSTN is in EC1 of SR, while in AF, it is mainly distributed in FB3 by analyzing the DEGs.

### Pseudotemporal ordering reveals branched cellular trajectory

**Fig. 6:**
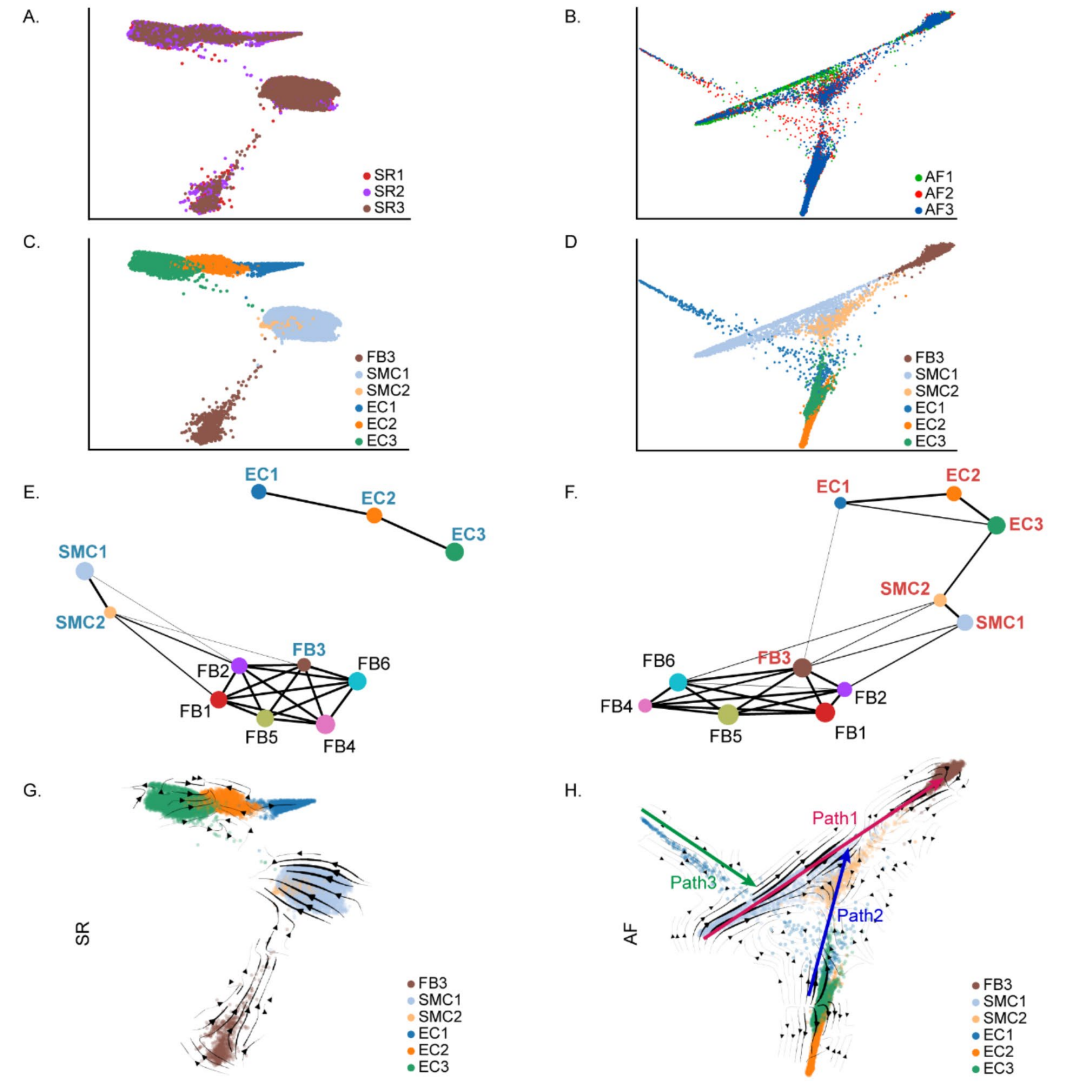
PAGA predicts developmental trajectories. **A**, **B.** PAGA graphs obtained after running PAGA on partitions corresponding to samples in SR and AF. **C**, **D.** PAGA graphs obtained after running PAGA on partitions corresponding to cell types in SR and AF. **E**, **F.** PAGA applied to scRNA-seq data of SR and AF. A PAGA graph is obtained by associating a node with each partition and connecting each node by weighted edges that represent a statistical measure of connectivity between partitions. **G**, **H.** PAGA embedding of cells with stochastic representation of the RNA velocity

To further explore the relationships of the FB3, SMCs, and ECs in AF, we researched the dynamic relationships between them using PAGA, which assesses the trajectories and connectivity of the diverse components of a manifold (Wolf et al. 2019). This result demonstrated that SMC2 and FB3 cells were intercommunicated in SR and that AF, SMC1, EC1, and FB3 cells were correlated with each other in AF versus SR (Fig. 6a-f).

We next executed an RNA velocity analysis in which spliced and unspliced mRNA counts were considered to predict the potential direction and speed of cell state transitions. This analysis distinguishes three sets of vectors, which are defined as paths. In AF, these analyses suggested that SMCs tended to differentiate to FBs, especially FB3 (path 1), and there is also differentiation between EC3 and SMC (path 2). Although there is a differentiated relationship between EC1 and FB3 (path 3), its role is weak. These findings were completely absent in SR (Fig. 6g and h). GO analyses suggested that cell migration, cell adhesion, ECM structural constituent, protein binding, collagen-containing ECM, and external encapsulating structure were enriched in paths (Figs. S5a, S5b).

Although path 3 differentiation was not obvious, we previously found changes in POSTN expression levels between EC1 and FB3 in SR and AF. Therefore, we analyzed the expression trends of POSTN transcription factors in EC1 and FB3 and found that the expression level of POSTN was higher in EC1 and FB3 (path 1). This further explains our previous findings that POSTN in EC1 likely play a key role in the pathologic process of AF (Fig. S5c).

Taken together, these results indicate that SMCs and ECs maybe more important in AF development than previously considered. We will further verify the differentiation and signaling pathways between SMCs, ECs, and FB3 using in vitro and in vivo experiments, which will provide new ideas and methods for the treatment of AF.

### Combining proteomics and GWAS to confirm the most relevant AF proteins, genes, and cell types

**Fig. 7:**
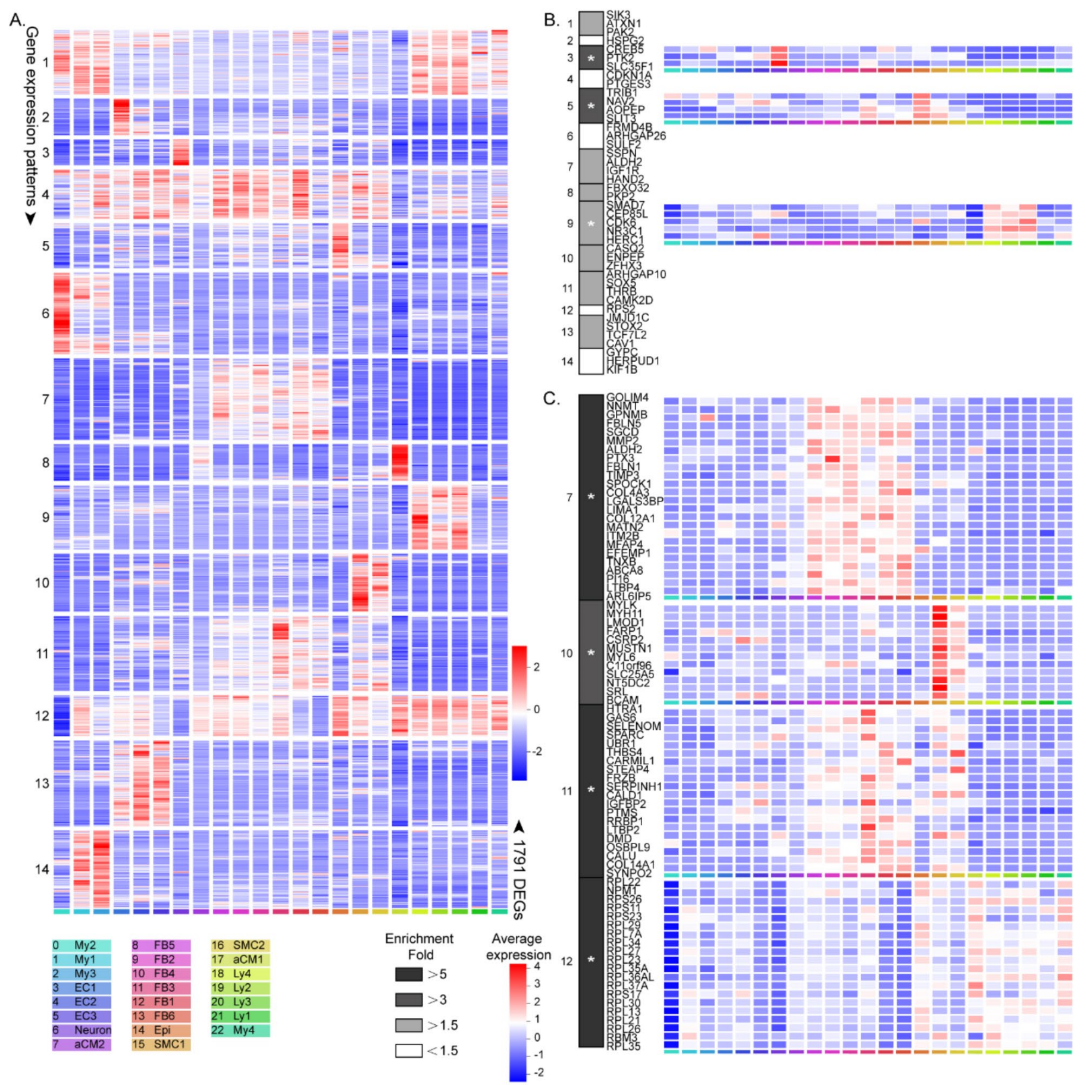
Projection of AF genome-wide association studies (GWAS) and proteomics–associated genes and proteins. **(A)** Heatmap of average expression of 1791 differentially expressed genes (DEGs) divided into 14 gene expression patters that best-matched cluster or cell type identity. **(B)** Enrichment of 42 AF GWAS associated genes across the 14 gene expression patterns. **(C)** Heatmap of average relative expression of significantly enriched AF proteins from gene expression pattern no. 7, no. 10, no. 11 and no. 12. Asterisk indicates significant enrichment. ^*^P < 0.05

Here we aimed to identify specific cell types that are highly expressed based on the genes and proteins associated with AF in GWAS and proteomics, thus efficiently identifying specific candidate genes and proteins for functional follow-up. According to the summary statistics of the last AF GWAS, there are 281 protein-coding genes (Roselli et al. 2018, Weng et al. 2017; Sakaue et al. 2021; Nielsen et al. 2018; Nielsen et al. 2018; Low et al. 2017; Lee et al. 2017; Larson et al. 2007; Kertai et al. 2015; Jiang et al. 2021; Hong et al. 2021; He et al. 2016; Gudbjartsson et al. 2009; Gudbjartsson et al. 2007; Ellinor et al. 2010; Ellinor et al. 2012; Christophersen et al. 2017; Benjamin et al. 2009) (Table S8); our proteomic results revealed that 625 proteins were differentially expressed between SR and AF (Figs. S6a-d; Table S7). Next, we chose the genes that would best represent each individual cell type by identifying DEGs. A total of 1791 DEGs were divided into 14 gene expression patterns that best matched the scRNA-seq populations (Fig. 7a). We overlapped the 281 AF-related genes and 625 proteins with the 1791 DEGs and obtained 42 gene and 139 protein overlaps.

We found a prominent enrichment of GWAS relevant genes in patterns 3, 5, and 9 (permutation over random data: *P* = 0.005, *P* = 0.008, and *P* = 0.01, respectively; Fig. 7b). Gene pattern 3 was related to higher expression in the neuron cluster (Fig. 7b) and consisted of *CREB5*, *PTK2*, and *SLC35F1*. Gene pattern 5 is characterized by gene expression in connection with epicardial cells and FB3 (Fig. 7b) and contained *TRIB1*, *NAV2*, *AOPEP*, and *SLIT3*. Gene pattern 9 is primarily related to higher expression in Ly subclusters 2, 3, and 4. This pattern contained SMAD7, *CEP85L*, *CDK6*, *NR3C1*, and *HERC1* (Fig. 7b).

In addition, a remarkable enrichment of proteomics-related proteins in patterns 7, 10, 11, and 12 (all permutation over random data, *P* = 0; Fig. 7c) was found. The genes in pattern 7 are linked with stronger expression in the FBs and consisted of *GOLIM4*, *NNMT*, *GPNMB*,*FBLN5*,*SGCD*,*MMP2*,*ALDH2*,*PTX3*,*FBLN1*, and *TIMP3* (Fig. 7c). Pattern 10 was marked with expression of genes associated with all two SMC populations, especially SMC1 and contained *MYLK*,*MYH11*,*LMOD1*,*FARP1*,*CSRP2*,*MUSTN1*,*MYL6*,*C11orf96*,*SLC25A5*,*NT5DC2*,*SRL*, and *BCAM* (Fig. 7c). Pattern 11 genes were mainly related to higher expressions in FB3. This pattern contained *HTRA1*,*GAS6*,*SPARC*,*FRZB*,*IGFBP2*, and *LTBP2*. On the contrary, the genes in pattern 12 were obviously concerned with lower expression in My2, and included *NPM1*, *RBM3*, and ribosomal proteins (Fig. 7c).

Interestingly, our results suggested that neuronal, epicardial, FB3, and Lys cells are an interesting initial point for functional testing by genes associated with AF in GWAS, but the proteomics results showed that AF susceptibility genes are closely related to FBs and SMCs. Notably, in patterns 2, 3, and 14, there was also a large accumulation of proteomic-related proteins (permutation over random data: *P* = 0.000440006, *P* = 0.007893439, and *P* = 0.0000533; Table S4). Genes in patterns 2, 3 and 14 were related to higher expression in EC1, neuron, and Mys cells, respectively. These patterns contained *INMT*,*TXNDC5*,*DKK3*,*SCN7A*,*MAP4*,*UBE2D1*,*LYZ*, and *EMILIN2* (Table S5).

## Discussion

The scRNA-seq technology has been used to specifically characterize the cellular landscape of the murine and human heart (Asp et al. 2019; Gladka 2021; Litvinukova et al. 2020; Molenaar and van Rooij 2018; Skelly et al. 2018; Tucker et al. 2020). Despite evidence of cellular heterogeneity in the heart, our understanding of cardiac homeostasis and disturbances remains limited by our understanding of cell type complexity and differences and specific functions of inter-cell type and subtype in the adult heart.

Our research brings insight into the cellular landscape and communication networks of the adult atrial from normal to diseased states (such as AF). Regarding single-cell sequencing, the precious nature of human data emphasizes the inherent technical and logistical challenges related to such studies, including isolating and preserving fresh tissue, isolating cells, and coordinating clinical and laboratory teams (Tucker et al. 2020). After meeting these demands, we utilized scRNA-seq to study all live cells in the human atria (right atrial appendages) of six samples and showed an extremely diverse cellular landscape consisting of eight cell clusters and 23 cell subclusters. The cell populations were similar to those reported by previous studies (Litvinukova et al. 2020), indicating our study’s reproducibility. However, we were missing mesothelial cells, pericytes, and adipocytes, probably since we used atrial tissue rather than whole heart single-cell sequencing. In addition, the adipocyte content was low, and no specific markers were observed. Notably, although we did not use pericytes, SMC2 highly expressed some pericytes markers, such as *RGS5* and *ABCC9*. Moreover, atrial tissue from another 12 patients were used for proteomics and mFISH testing (proteomics: six patients; mFISH: six patients).

Myofibroblasts are reportedly increased in density and essential to extracellular matrix (ECM) production in fibrotic diseases (Henderson et al. 2020). Therefore, FB3 may be a myofibroblast that play an essential role in AF development. A study (Zhang et al. 2021) concluded that ECs do not transdifferentiate into myofibroblasts and do not transiently express some known mesenchymal genes during homeostasis and fibrosis in the adult human heart. Resident fibroblasts, which are converted into myofibroblasts by the activation of mesenchymal gene expression, are the main factors leading to cardiac fibrosis. Therefore, our results suggested that FB2 and FB4 may be cardiac resident fibroblasts involved in the process of fibrosis.

Here we revealed cell–cell communication between SMCs and FBs and their alterations in AF progression. Our results observed that cell communication between SMC and FBs is largely similar at the time of AF occurrence, and their input and output signals belong to the same pattern, including ANGPTL, PDGF, HSPG and PROS signaling pathways. Pseudotime and RNA velocity analyses also indicated that SMCs were at an early stage of differentiation and could be transdifferentiated into FB3. Meanwhile, a study (Harlaar et al. 2022) reported that inserting an oncogene into the DNA of heart muscle cells can control the “opening” and “closing” of cardiac muscle cells, allowing them to rapidly proliferate. Therefore, oncogenes likely played a key role in the present study, which found that SMC2 specifically highly expressed some oncogenes, including *FRMD3*, *GUCY1A2*, and *INPP4B*, in patients of AF, but the detailed roles of oncogenes in the pathological process of AF deserves further exploration.

We also observed that the proportion of FB3 cells was significantly increased in the AF, and that fibrosis-related genes, including *COL3A1*, *ELN*, and *ACTA2*, were highly expressed. GO clustering analysis is related to collagen-containing ECM and ECM structural constituents. The pathological mechanism of myocardial fibrosis is associated with changes in the cellular and neurohumoral environment resulting in alterations in fibroblast activity and ECM turnover (Berk et al. 2007; Fredj et al. 2005; Lucas et al. 2010). As a novel mediator of cardiac fibrosis, the endoplasmic reticulum (ER) protein TXNDC5 is enriched in cardiac fibroblasts (Shih et al. 2018). That study suggested that TXNDC5 promotes cardiac fibrosis primarily through redox-dependent cardiac fibroblast activation and enhances ECM production by boosting ECM protein folding (Shih et al. 2018). The excessive accumulation of ECM caused by myocardial fibrosis will impair the systolic function of the heart and lead to arrhythmia. our study found that the fibroblast-enriched ER protein TXNDC5 was increased in EC1 cells of AF. Therefore, it is possible that TXNDC5 are involved in the process of ECM protein folding, which will lead to atrial fibrosis.

The secreted 90-kDa POSTN, a nonstructural component of the ECM, is a crucial stromal cytokine in cardiac mesenchymal tissue. Furthermore, the mediating role of POSTN in ECM crosstalk has received attention due to its correlation with fibroproliferative cardiac diseases (Landry et al. 2018). Consistent with our findings, examination of the atrial appendage of AF patients with elective valve replacement surgery showed a strong positive association between the expression level of POSTN and the degree of atrial fibrosis (Wu et al. 2015). One of the most interesting discoveries in the current study was that POSTN was significantly increased in FB3 cells of the AF group versus EC1 cells of the SR group. Our finding suggests that EC1 may play a crucial role in collagen deposition of FB3 in AF through POSTN. The expression traces along trajectories of POSTN in path 3 and the intercellular communication between ECs and FBs through the PERIOSTIN signaling pathway also confirmed the above results.

Some studies found that, after myocardial injury ECs are transformed into mesenchymal cells, mainly fibroblasts, and that pathological fibrosis is significantly promoted by the endothelial to mesenchymal transition (LeBleu et al. 2013; Ranchoux et al. 2015; Widyantoro et al. 2010; Xu et al. 2015; Zeisberg et al. 2007). Zhang et al. (Zhang et al. 2021) reported that, during cardiac fibrosis, adult ECs do not transdifferentiate into myofibroblasts or transiently express genes for the endothelial to mesenchymal transition. Our results suggest that fibrosis may be mainly caused by *TXNDC5* and *POSTN* in ECs.

GWAS have demonstrated remarkable ability to discover novel disease susceptibility genes and biological processes and translate these findings into clinical care (Tam et al. 2019). A great challenge following GWAS screening for disease susceptibility genes and loci is identifying candidate genes and signaling pathways with clinical potential (Boyle et al. 2017). Here we created genetic profiling based on AF susceptibility genes from GWAS and used single-cell resolution expression in human atrial tissue (right atrial appendages) to identify possible targets for future functional follow-up. We also mapped the differential protein genes obtained by proteomics to cell types. We precisely concentrated on common variants at risk loci and protein-coding genes associated with AF that matched the cell populations. Studies have built proteo-genomic linkages within and between diseases and affirm the significance of cis-protein variants for annotation of possibly disease-causing genes at loci identified in GWAS investigations, thereby addressing a stumbling block to experimental verification and clinical translation of genes discovered (Pietzner et al. 2021). Therefore, we mapped proteomic differential proteins to cell types to explore whether genes related to AF susceptibility could be further identified. Genes are more directly linked to disease through encoded proteins, thereby facilitating the accelerated identification of drug targets.

## Conclusion

Overall, our study provided a variety of reference data regarding the numerous cell types and interaction networks in the adult human atrial (right atrial appendages) as well as a useful tool to support research into the underlying causes of AF and identify new therapeutic targets. We recognize the limitations of sample size, gender imbalance and cell capture from different sources, and unexpected biases in surgical sampling. In addition, considering the requirements of experimental ethics and the interests of patients, only the right atrial appendages of patients were studied. However, we hope that our results will inform the study of AF, advance clinical and experimental research, and provide important insights into understanding the mechanisms of AF.

## Electronic supplementary material

Below is the link to the electronic supplementary material.

Supplementary Material 1

Supplementary Material 2

Supplementary Material 3

Supplementary Material 4

Supplementary Material 5

Supplementary Material 6

Supplementary Material 7

Supplementary Material 8

Supplementary Material 9

Supplementary Material 10

## Data Availability

The datasets used and/or analyzed during the current study available from the corresponding author on reasonable request.
