# Supplementary material for: Single-cell RNA sequencing reveals the contribution of smooth muscle cells and endothelial cells to fibrosis in human atrial tissue with atrial fibrillation
Source: Mol Med. 2024 Dec 19;30:247. doi: 10.1186/s10020-024-00999-1 (PMC11661033; doi:10.1186/s10020-024-00999-1)
Supplement: Supplementary file 1 — Supplementary Material 1 [file 10020_2024_999_MOESM1_ESM.docx]

**Supplementary Methods**

**Methods**

***Single-cell RNA sequence data processing***

Raw FASTQ files were mapped to the reference human genome using Cell Ranger 4.0 (https://support.10xgenomics.com/single-cell-gene-expression/software/downloads/), which performs alignment, filtering, barcode counting, and UMI counting. Each sample was aligned to human genome (GRCh38, provided by 10× Genomics) by the ‘Cell Ranger Count’ function to obtain the raw gene expression matrices. These matrices were further analyzed by Scanpy (Wolf, Angerer, and Theis 2018) version 1.91 for quality control and downstream analysis. In this manner, cell-gene UMI counting matrices for downstream analysis were produced. Cells having a mitochondrial transcription ratio greater than 5%, more than 6,000 identified genes, or fewer than 200 genes were eliminated from each sample. Cell doublets (occasional pairs of cells that are not dissociated during sample preparation) were identified using the scrublet(Wolock, Lopez, and Klein 2019) tool and then discarded. After quality control, a total of 71,440 cells were retained. The SCTransform function in the Seurat package was used to standardize the gene expression levels in each cell(Stuart et al. 2019). Batch effects derived from technical and biological covariates, including the batch and harvested time, were corrected using harmonypy(Korsunsky et al. 2019) version 0.0.5. Based on their average expression and dispersion, we classified the top 2000 most variable genes as highly variable genes (HVG). By running the principal component analysis (PCA) on the HVG, we were able to reduce the dimensionality of the data. After constructing the closest neighbor network, the nearest neighbor clustering of the PCA reduced data with the Louvain algorithm was utilized to divide the cells. The clusters that resulted from uniform approximation and projection were seen in a 2-D embedding (UMAP, version 0.4.6). Finally, the expression matrix was given the coordinates of the UMAP and cluster tags as a result for downstream analysis on each cell.

***Cell–cell communication analysis.***

We utilized CellChat 2.0(Jin et al. 2021), a program that can quantitatively predict and evaluate intercellular communication networks from single-cell RNA-sequencing (scRNA-seq) data, to find potential linkages between and within each cell cluster to all other cells.

***Myocardial histopathology***

The atrial tissue was fixed in 4% paraformaldehyde, and serially sectioned. The sections were dyed with hematoxylin-eosin (HE) staining, Masson's trichrome stain, and Sirius red dye. The hearts of three human were stained and analyzed in each group. Finally, ImageJ software was used for quantitative analysis of images.

***FISH with RNAscope probes.***

Fresh tissue was fixed in neutral-buffered 4% paraformaldehyde (contains RNAase-free ddH2O) to produce formalin-fixed paraffin-embedded (FFPE) samples for 18–36 hours before being embedded in paraffin blocks. The slicer, the piece of the slice machine, and the 62 ℃ oven roast the paraffin for two hours. Dehydration and waxing follow. To achieve goals, add proteinase K (20 ug/ml) working solution. To each portion, add the pre-hybridization solution, and incubate for 1 hour at 37 °C. Add the probe hybridization solution, washing, and removal of the pre-hybridization solution. All nuclei were stained using DAPI. Channels: DAPI glows blue by UV excitation wavelength 330-380 nm and emission wavelength 420 nm; FAM glows green by excitation wavelength 465-495 nm and emission wavelength 515-555 nm; CY3 glows red by excitation wavelength 510-560 nm and emission wavelength 590 nm. Data analysis was performed using ImageJ.

***Proteomics.***

After being extracted from the liquid nitrogen, the left atrial tissue was put into tubes with low protein binding and lysed with 1 mM phenylmethylsulfonyl fluoride (PMSF). After that, samples were further lysed using ultrasound. The samples were sonicated, then centrifuged at 12,000 g for 10 min, collecting the supernatant. Then it was done once more. Bicinchoninic acid (BCA) protein assay(Smith et al. 1985) was used to measure the protein concentration, and samples were kept at 80 °C. Following that, 12% SDS-PAGE was used to isolate each protein sample. The gel was then dyed using Coomassie Brilliant Blue in accordance with the Candiano technique(Candiano et al. 2004). The trypsinization and labeling of some proteins was followed by mixing the same quantity of each labeled sample and running it through chromatography. Utilizing SOLA^TM^ SPE 96-well plates, the digested peptides were desalted. Liquid chromatography-tandem mass spectrometry (LC-MS/MS) was then applied to the sample. LC-MS/MS raw files imported into Spectronaut Pulsar for library search and building. Processing of DIA's raw data is done using Spectronaut Pulsar software.

***Differential expression analysis***

MAST(Finak et al. 2015) version 1.14.0 was used to identify DEGs in the donors compared to the control in each cell type, which fit a hurdle model to the expression of each gene and performed zero-inflated regression analysis. The discrete and continuous coefficients of the model were retrieved, *P* values were calculated using the likelihood ratio test, and *q* values were individually assessed using the Benjamini-Hochberg correction for each cell subset comparison.

***PAGA analysis***

We quantified the connectivity of cell clusters using the PAGA method(Wolf et al. 2019), as implemented in the scRNA-seq analysis package of Scanpy. This computational process was carried out on the same subset of variable genes as for clustering.

***Velocity analysis***

ScVelo(Bergen et al. 2020) version 0.2.4 was used to infer transitional states for individual cells. Specifically, count matrices of unspliced and spliced abundance were obtained using the velocyto(La Manno et al. 2018) version 0.17 pipeline. Then, the matrices were input to scVelo for normalization, log-transformation and calculation of first- and second-order moments for each cell across its nearest neighbors. Next, the velocities for each cell were computed, and the velocity graph was constructed using the *tl.velocity()* function with the ‘stochastic’ mode. The latent time representing the internal clock of cells was obtained using the *tl.latent_time()* function with default parameters.

***Differential gene expression analysis and mapping of GWAS loci and proteomics.***

For the analysis and mapping of AF associated gene to the single-cell RNAseq data, we applied three steps: 1) we processed genes based on the AF GWAS summary statistics(Roselli et al. 2018) and got differential proteins from proteomics, 2) we determined the genes and proteins differentially expressed between cell type and therefore specific to these cell types, and 3) we overlapped the GWAS-derived genes as well as differential proteins and differentially expressed genes to identify genes associated to AF and specific to a cell type.

Step 1: We used the summary statistics from the GWAS on AF(Roselli et al. 2018, Weng et al. 2017, Sakaue et al. 2021, Nielsen, Thorolfsdottir, et al. 2018, Nielsen, Fritsche, et al. 2018, Low et al. 2017, Lee et al. 2017, Larson et al. 2007, Kertai et al. 2015, Jiang et al. 2021, Hong et al. 2021, He et al. 2016, Gudbjartsson et al. 2009, Gudbjartsson et al. 2007, Ellinor et al. 2010, Ellinor et al. 2012, Christophersen et al. 2017, Benjamin et al. 2009).

Step 2: For the single-cell analysis and mapping, we have focused only on genes that 1) 1) one cell cluster express the gene with absolute value of logFC of >= 0.5, and 2) the gene passes the Bonferroni adjusted significance threshold of p.adjusted < 0.05 for this test. The 1791 DEGs were sorted into 14 gene expression patterns by K-means clustering based on average expression per cell type (Table S6).

Step 3: After this, the 281 AF associated genes derived from the GWAS summary statistics as well as 625 proteins and the 1791 DEGs derived from the scRNAseq data are overlapped. This resulted in an overlap of 42 genes and 139 proteins, respectively that are both highly expressed in scRNAseq data (a DEG). We calculated enrichment of the overlap DEGs in the 14 gene expression patterns using permutation analysis. We then calculated positive enrichment for each gene expression pattern for the mapped GWAS genes compared to the random sets and determined the enrichment of DEGs. Downstream processing was performed using custom python scripts. Heatmap and plots were made with seaborn package.

***Overrepresentation test for Gene Ontology (GO) terms***

An overrepresentation test for DEGs from each cell type was run using g:Profiler(Raudvere et al. 2019). The background was comprised of all genes examined for differential expression in a certain cluster. Enriched pathways were those with an FDR<0.05.

***Estimation of the cell proportion***

The calculation of the relevance of variations in each cell type's proportion was done as previously mentioned(Haber et al. 2017). In a summary, the Poisson process was used to model the detected number of each cell type as a random count variable. The total number of captured cells in a specific sample was given as an offset variable, and the condition of each sample was given as a covariate, in order to model the rate of detection.

## References

Benjamin, E. J., K. M. Rice, D. E. Arking, A. Pfeufer, C. van Noord, A. V. Smith, R. B. Schnabel, J. C. Bis, E. Boerwinkle, M. F. Sinner, A. Dehghan, S. A. Lubitz, R. B. D'Agostino, Sr., T. Lumley, G. B. Ehret, J. Heeringa, T. Aspelund, C. Newton-Cheh, M. G. Larson, K. D. Marciante, E. Z. Soliman, F. Rivadeneira, T. J. Wang, G. Eiriksdottir, D. Levy, B. M. Psaty, M. Li, A. M. Chamberlain, A. Hofman, R. S. Vasan, T. B. Harris, J. I. Rotter, W. H. Kao, S. K. Agarwal, B. H. Stricker, K. Wang, L. J. Launer, N. L. Smith, A. Chakravarti, A. G. Uitterlinden, P. A. Wolf, N. Sotoodehnia, A. Kottgen, C. M. van Duijn, T. Meitinger, M. Mueller, S. Perz, G. Steinbeck, H. E. Wichmann, K. L. Lunetta, S. R. Heckbert, V. Gudnason, A. Alonso, S. Kaab, P. T. Ellinor, and J. C. Witteman. 2009. "Variants in ZFHX3 are associated with atrial fibrillation in individuals of European ancestry." *Nat Genet* 41 (8):879-81. doi: 10.1038/ng.416.

Bergen, V., M. Lange, S. Peidli, F. A. Wolf, and F. J. Theis. 2020. "Generalizing RNA velocity to transient cell states through dynamical modeling." *Nat Biotechnol*. doi: 10.1038/s41587-020-0591-3.

Candiano, G., M. Bruschi, L. Musante, L. Santucci, G. M. Ghiggeri, B. Carnemolla, P. Orecchia, L. Zardi, and P. G. Righetti. 2004. "Blue silver: a very sensitive colloidal Coomassie G-250 staining for proteome analysis." *Electrophoresis* 25 (9):1327-33. doi: 10.1002/elps.200305844.

Christophersen, I. E., M. Rienstra, C. Roselli, X. Yin, B. Geelhoed, J. Barnard, H. Lin, D. E. Arking, A. V. Smith, C. M. Albert, M. Chaffin, N. R. Tucker, M. Li, D. Klarin, N. A. Bihlmeyer, S. K. Low, P. E. Weeke, M. Muller-Nurasyid, J. G. Smith, J. A. Brody, M. N. Niemeijer, M. Dorr, S. Trompet, J. Huffman, S. Gustafsson, C. Schurmann, M. E. Kleber, L. P. Lyytikainen, I. Seppala, R. Malik, Arvr Horimoto, M. Perez, J. Sinisalo, S. Aeschbacher, S. Theriault, J. Yao, F. Radmanesh, S. Weiss, A. Teumer, S. H. Choi, L. C. Weng, S. Clauss, R. Deo, D. J. Rader, S. H. Shah, A. Sun, J. C. Hopewell, S. Debette, G. Chauhan, Q. Yang, B. B. Worrall, G. Pare, Y. Kamatani, Y. P. Hagemeijer, N. Verweij, J. E. Siland, M. Kubo, J. D. Smith, D. R. Van Wagoner, J. C. Bis, S. Perz, B. M. Psaty, P. M. Ridker, J. W. Magnani, T. B. Harris, L. J. Launer, M. B. Shoemaker, S. Padmanabhan, J. Haessler, T. M. Bartz, M. Waldenberger, P. Lichtner, M. Arendt, J. E. Krieger, M. Kahonen, L. Risch, A. J. Mansur, A. Peters, B. H. Smith, L. Lind, S. A. Scott, Y. Lu, E. B. Bottinger, J. Hernesniemi, C. M. Lindgren, J. A. Wong, J. Huang, M. Eskola, A. P. Morris, I. Ford, A. P. Reiner, G. Delgado, L. Y. Chen, Y. I. Chen, R. K. Sandhu, M. Li, E. Boerwinkle, L. Eisele, L. Lannfelt, N. Rost, C. D. Anderson, K. D. Taylor, A. Campbell, P. K. Magnusson, D. Porteous, L. J. Hocking, E. Vlachopoulou, N. L. Pedersen, K. Nikus, M. Orho-Melander, A. Hamsten, J. Heeringa, J. C. Denny, J. Kriebel, D. Darbar, C. Newton-Cheh, C. Shaffer, P. W. Macfarlane, S. Heilmann-Heimbach, P. Almgren, P. L. Huang, N. Sotoodehnia, E. Z. Soliman, A. G. Uitterlinden, A. Hofman, O. H. Franco, U. Volker, K. H. Jockel, M. F. Sinner, H. J. Lin, X. Guo, Metastroke Consortium of the ISGC, Charge Consortium Neurology Working Group of the, M. Dichgans, E. Ingelsson, C. Kooperberg, O. Melander, R. J. F. Loos, J. Laurikka, D. Conen, J. Rosand, P. van der Harst, M. L. Lokki, S. Kathiresan, A. Pereira, J. W. Jukema, C. Hayward, J. I. Rotter, W. Marz, T. Lehtimaki, B. H. Stricker, M. K. Chung, S. B. Felix, V. Gudnason, A. Alonso, D. M. Roden, S. Kaab, D. I. Chasman, S. R. Heckbert, E. J. Benjamin, T. Tanaka, K. L. Lunetta, S. A. Lubitz, P. T. Ellinor, and A. FGen Consortium. 2017. "Large-scale analyses of common and rare variants identify 12 new loci associated with atrial fibrillation." *Nat Genet* 49 (6):946-952. doi: 10.1038/ng.3843.

Ellinor, P. T., K. L. Lunetta, C. M. Albert, N. L. Glazer, M. D. Ritchie, A. V. Smith, D. E. Arking, M. Muller-Nurasyid, B. P. Krijthe, S. A. Lubitz, J. C. Bis, M. K. Chung, M. Dorr, K. Ozaki, J. D. Roberts, J. G. Smith, A. Pfeufer, M. F. Sinner, K. Lohman, J. Ding, N. L. Smith, J. D. Smith, M. Rienstra, K. M. Rice, D. R. Van Wagoner, J. W. Magnani, R. Wakili, S. Clauss, J. I. Rotter, G. Steinbeck, L. J. Launer, R. W. Davies, M. Borkovich, T. B. Harris, H. Lin, U. Volker, H. Volzke, D. J. Milan, A. Hofman, E. Boerwinkle, L. Y. Chen, E. Z. Soliman, B. F. Voight, G. Li, A. Chakravarti, M. Kubo, U. B. Tedrow, L. M. Rose, P. M. Ridker, D. Conen, T. Tsunoda, T. Furukawa, N. Sotoodehnia, S. Xu, N. Kamatani, D. Levy, Y. Nakamura, B. Parvez, S. Mahida, K. L. Furie, J. Rosand, R. Muhammad, B. M. Psaty, T. Meitinger, S. Perz, H. E. Wichmann, J. C. Witteman, W. H. Kao, S. Kathiresan, D. M. Roden, A. G. Uitterlinden, F. Rivadeneira, B. McKnight, M. Sjogren, A. B. Newman, Y. Liu, M. H. Gollob, O. Melander, T. Tanaka, B. H. Stricker, S. B. Felix, A. Alonso, D. Darbar, J. Barnard, D. I. Chasman, S. R. Heckbert, E. J. Benjamin, V. Gudnason, and S. Kaab. 2012. "Meta-analysis identifies six new susceptibility loci for atrial fibrillation." *Nat Genet* 44 (6):670-5. doi: 10.1038/ng.2261.

Ellinor, P. T., K. L. Lunetta, N. L. Glazer, A. Pfeufer, A. Alonso, M. K. Chung, M. F. Sinner, P. I. de Bakker, M. Mueller, S. A. Lubitz, E. Fox, D. Darbar, N. L. Smith, J. D. Smith, R. B. Schnabel, E. Z. Soliman, K. M. Rice, D. R. Van Wagoner, B. M. Beckmann, C. van Noord, K. Wang, G. B. Ehret, J. I. Rotter, S. L. Hazen, G. Steinbeck, A. V. Smith, L. J. Launer, T. B. Harris, S. Makino, M. Nelis, D. J. Milan, S. Perz, T. Esko, A. Kottgen, S. Moebus, C. Newton-Cheh, M. Li, S. Mohlenkamp, T. J. Wang, W. H. Kao, R. S. Vasan, M. M. Nothen, C. A. MacRae, B. H. Stricker, A. Hofman, A. G. Uitterlinden, D. Levy, E. Boerwinkle, A. Metspalu, E. J. Topol, A. Chakravarti, V. Gudnason, B. M. Psaty, D. M. Roden, T. Meitinger, H. E. Wichmann, J. C. Witteman, J. Barnard, D. E. Arking, E. J. Benjamin, S. R. Heckbert, and S. Kaab. 2010. "Common variants in KCNN3 are associated with lone atrial fibrillation." *Nat Genet* 42 (3):240-4. doi: 10.1038/ng.537.

Finak, G., A. McDavid, M. Yajima, J. Deng, V. Gersuk, A. K. Shalek, C. K. Slichter, H. W. Miller, M. J. McElrath, M. Prlic, P. S. Linsley, and R. Gottardo. 2015. "MAST: a flexible statistical framework for assessing transcriptional changes and characterizing heterogeneity in single-cell RNA sequencing data." *Genome Biol* 16:278. doi: 10.1186/s13059-015-0844-5.

Gudbjartsson, D. F., D. O. Arnar, A. Helgadottir, S. Gretarsdottir, H. Holm, A. Sigurdsson, A. Jonasdottir, A. Baker, G. Thorleifsson, K. Kristjansson, A. Palsson, T. Blondal, P. Sulem, V. M. Backman, G. A. Hardarson, E. Palsdottir, A. Helgason, R. Sigurjonsdottir, J. T. Sverrisson, K. Kostulas, M. C. Ng, L. Baum, W. Y. So, K. S. Wong, J. C. Chan, K. L. Furie, S. M. Greenberg, M. Sale, P. Kelly, C. A. MacRae, E. E. Smith, J. Rosand, J. Hillert, R. C. Ma, P. T. Ellinor, G. Thorgeirsson, J. R. Gulcher, A. Kong, U. Thorsteinsdottir, and K. Stefansson. 2007. "Variants conferring risk of atrial fibrillation on chromosome 4q25." *Nature* 448 (7151):353-7. doi: 10.1038/nature06007.

Gudbjartsson, D. F., H. Holm, S. Gretarsdottir, G. Thorleifsson, G. B. Walters, G. Thorgeirsson, J. Gulcher, E. B. Mathiesen, I. Njolstad, A. Nyrnes, T. Wilsgaard, E. M. Hald, K. Hveem, C. Stoltenberg, G. Kucera, T. Stubblefield, S. Carter, D. Roden, M. C. Ng, L. Baum, W. Y. So, K. S. Wong, J. C. Chan, C. Gieger, H. E. Wichmann, A. Gschwendtner, M. Dichgans, G. Kuhlenbaumer, K. Berger, E. B. Ringelstein, S. Bevan, H. S. Markus, K. Kostulas, J. Hillert, S. Sveinbjornsdottir, E. M. Valdimarsson, M. L. Lochen, R. C. Ma, D. Darbar, A. Kong, D. O. Arnar, U. Thorsteinsdottir, and K. Stefansson. 2009. "A sequence variant in ZFHX3 on 16q22 associates with atrial fibrillation and ischemic stroke." *Nat Genet* 41 (8):876-8. doi: 10.1038/ng.417.

Haber, A. L., M. Biton, N. Rogel, R. H. Herbst, K. Shekhar, C. Smillie, G. Burgin, T. M. Delorey, M. R. Howitt, Y. Katz, I. Tirosh, S. Beyaz, D. Dionne, M. Zhang, R. Raychowdhury, W. S. Garrett, O. Rozenblatt-Rosen, H. N. Shi, O. Yilmaz, R. J. Xavier, and A. Regev. 2017. "A single-cell survey of the small intestinal epithelium." *Nature* 551 (7680):333-339. doi: 10.1038/nature24489.

He, L., Y. Kernogitski, I. Kulminskaya, Y. Loika, K. G. Arbeev, E. Loiko, O. Bagley, M. Duan, A. Yashkin, S. V. Ukraintseva, M. Kovtun, A. I. Yashin, and A. M. Kulminski. 2016. "Pleiotropic Meta-Analyses of Longitudinal Studies Discover Novel Genetic Variants Associated with Age-Related Diseases." *Front Genet* 7:179. doi: 10.3389/fgene.2016.00179.

Hong, M., Y. Ebana, J. Shim, E. K. Choi, H. E. Lim, I. Hwang, H. T. Yu, T. H. Kim, J. S. Uhm, B. Joung, S. Oh, M. H. Lee, Y. H. Kim, S. H. Jee, and H. N. Pak. 2021. "Ethnic similarities in genetic polymorphisms associated with atrial fibrillation: Far East Asian vs European populations." *Eur J Clin Invest* 51 (9):e13584. doi: 10.1111/eci.13584.

Jiang, L., Z. Zheng, H. Fang, and J. Yang. 2021. "A generalized linear mixed model association tool for biobank-scale data." *Nat Genet* 53 (11):1616-1621. doi: 10.1038/s41588-021-00954-4.

Jin, Suoqin, Christian F. Guerrero-Juarez, Lihua Zhang, Ivan Chang, Raul Ramos, Chen-Hsiang Kuan, Peggy Myung, Maksim V. Plikus, and Qing Nie. 2021. "Inference and analysis of cell-cell communication using CellChat." *Nature Communications* 12 (1):1088. doi: 10.1038/s41467-021-21246-9.

Kertai, M. D., Y. J. Li, Y. Ji, W. Qi, F. W. Lombard, S. H. Shah, W. E. Kraus, M. Stafford-Smith, M. F. Newman, C. A. Milano, N. Waldron, M. V. Podgoreanu, J. P. Mathew, Genetics Duke Perioperative, and Team Safety Outcomes Investigative. 2015. "Genome-wide association study of new-onset atrial fibrillation after coronary artery bypass grafting surgery." *Am Heart J* 170 (3):580-90 e28. doi: 10.1016/j.ahj.2015.06.009.

Korsunsky, I., N. Millard, J. Fan, K. Slowikowski, F. Zhang, K. Wei, Y. Baglaenko, M. Brenner, P. R. Loh, and S. Raychaudhuri. 2019. "Fast, sensitive and accurate integration of single-cell data with Harmony." *Nat Methods* 16 (12):1289-1296. doi: 10.1038/s41592-019-0619-0.

La Manno, G., R. Soldatov, A. Zeisel, E. Braun, H. Hochgerner, V. Petukhov, K. Lidschreiber, M. E. Kastriti, P. Lonnerberg, A. Furlan, J. Fan, L. E. Borm, Z. Liu, D. van Bruggen, J. Guo, X. He, R. Barker, E. Sundstrom, G. Castelo-Branco, P. Cramer, I. Adameyko, S. Linnarsson, and P. V. Kharchenko. 2018. "RNA velocity of single cells." *Nature* 560 (7719):494-498. doi: 10.1038/s41586-018-0414-6.

Larson, M. G., L. D. Atwood, E. J. Benjamin, L. A. Cupples, R. B. D'Agostino, Sr., C. S. Fox, D. R. Govindaraju, C. Y. Guo, N. L. Heard-Costa, S. J. Hwang, J. M. Murabito, C. Newton-Cheh, C. J. O'Donnell, S. Seshadri, R. S. Vasan, T. J. Wang, P. A. Wolf, and D. Levy. 2007. "Framingham Heart Study 100K project: genome-wide associations for cardiovascular disease outcomes." *BMC Med Genet* 8 Suppl 1:S5. doi: 10.1186/1471-2350-8-S1-S5.

Lee, J. Y., T. H. Kim, P. S. Yang, H. E. Lim, E. K. Choi, J. Shim, E. Shin, J. S. Uhm, J. S. Kim, B. Joung, S. Oh, M. H. Lee, Y. H. Kim, and H. N. Pak. 2017. "Korean atrial fibrillation network genome-wide association study for early-onset atrial fibrillation identifies novel susceptibility loci." *Eur Heart J* 38 (34):2586-2594. doi: 10.1093/eurheartj/ehx213.

Low, S. K., A. Takahashi, Y. Ebana, K. Ozaki, I. E. Christophersen, P. T. Ellinor, A. FGen Consortium, S. Ogishima, M. Yamamoto, M. Satoh, M. Sasaki, T. Yamaji, M. Iwasaki, S. Tsugane, K. Tanaka, M. Naito, K. Wakai, H. Tanaka, T. Furukawa, M. Kubo, K. Ito, Y. Kamatani, and T. Tanaka. 2017. "Identification of six new genetic loci associated with atrial fibrillation in the Japanese population." *Nat Genet* 49 (6):953-958. doi: 10.1038/ng.3842.

Nielsen, J. B., L. G. Fritsche, W. Zhou, T. M. Teslovich, O. L. Holmen, S. Gustafsson, M. E. Gabrielsen, E. M. Schmidt, R. Beaumont, B. N. Wolford, M. Lin, C. M. Brummett, M. H. Preuss, L. Refsgaard, E. P. Bottinger, S. E. Graham, I. Surakka, Y. Chu, A. H. Skogholt, H. Dalen, A. P. Boyle, H. Oral, T. J. Herron, J. Kitzman, J. Jalife, J. H. Svendsen, M. S. Olesen, I. Njolstad, M. L. Lochen, A. Baras, O. Gottesman, A. Marcketta, C. O'Dushlaine, M. D. Ritchie, T. Wilsgaard, R. J. F. Loos, T. M. Frayling, M. Boehnke, E. Ingelsson, D. J. Carey, F. E. Dewey, H. M. Kang, G. R. Abecasis, K. Hveem, and C. J. Willer. 2018. "Genome-wide Study of Atrial Fibrillation Identifies Seven Risk Loci and Highlights Biological Pathways and Regulatory Elements Involved in Cardiac Development." *Am J Hum Genet* 102 (1):103-115. doi: 10.1016/j.ajhg.2017.12.003.

Nielsen, J. B., R. B. Thorolfsdottir, L. G. Fritsche, W. Zhou, M. W. Skov, S. E. Graham, T. J. Herron, S. McCarthy, E. M. Schmidt, G. Sveinbjornsson, I. Surakka, M. R. Mathis, M. Yamazaki, R. D. Crawford, M. E. Gabrielsen, A. H. Skogholt, O. L. Holmen, M. Lin, B. N. Wolford, R. Dey, H. Dalen, P. Sulem, J. H. Chung, J. D. Backman, D. O. Arnar, U. Thorsteinsdottir, A. Baras, C. O'Dushlaine, A. G. Holst, X. Wen, W. Hornsby, F. E. Dewey, M. Boehnke, S. Kheterpal, B. Mukherjee, S. Lee, H. M. Kang, H. Holm, J. Kitzman, J. A. Shavit, J. Jalife, C. M. Brummett, T. M. Teslovich, D. J. Carey, D. F. Gudbjartsson, K. Stefansson, G. R. Abecasis, K. Hveem, and C. J. Willer. 2018. "Biobank-driven genomic discovery yields new insight into atrial fibrillation biology." *Nat Genet* 50 (9):1234-1239. doi: 10.1038/s41588-018-0171-3.

Raudvere, U., L. Kolberg, I. Kuzmin, T. Arak, P. Adler, H. Peterson, and J. Vilo. 2019. "g:Profiler: a web server for functional enrichment analysis and conversions of gene lists (2019 update)." *Nucleic Acids Res* 47 (W1):W191-W198. doi: 10.1093/nar/gkz369.

Roselli, C., M. D. Chaffin, L. C. Weng, S. Aeschbacher, G. Ahlberg, C. M. Albert, P. Almgren, A. Alonso, C. D. Anderson, K. G. Aragam, D. E. Arking, J. Barnard, T. M. Bartz, E. J. Benjamin, N. A. Bihlmeyer, J. C. Bis, H. L. Bloom, E. Boerwinkle, E. B. Bottinger, J. A. Brody, H. Calkins, A. Campbell, T. P. Cappola, J. Carlquist, D. I. Chasman, L. Y. Chen, Y. I. Chen, E. K. Choi, S. H. Choi, I. E. Christophersen, M. K. Chung, J. W. Cole, D. Conen, J. Cook, H. J. Crijns, M. J. Cutler, S. M. Damrauer, B. R. Daniels, D. Darbar, G. Delgado, J. C. Denny, M. Dichgans, M. Dorr, E. A. Dudink, S. C. Dudley, N. Esa, T. Esko, M. Eskola, D. Fatkin, S. B. Felix, I. Ford, O. H. Franco, B. Geelhoed, R. P. Grewal, V. Gudnason, X. Guo, N. Gupta, S. Gustafsson, R. Gutmann, A. Hamsten, T. B. Harris, C. Hayward, S. R. Heckbert, J. Hernesniemi, L. J. Hocking, A. Hofman, Arvr Horimoto, J. Huang, P. L. Huang, J. Huffman, E. Ingelsson, E. G. Ipek, K. Ito, J. Jimenez-Conde, R. Johnson, J. W. Jukema, S. Kaab, M. Kahonen, Y. Kamatani, J. P. Kane, A. Kastrati, S. Kathiresan, P. Katschnig-Winter, M. Kavousi, T. Kessler, B. L. Kietselaer, P. Kirchhof, M. E. Kleber, S. Knight, J. E. Krieger, M. Kubo, L. J. Launer, J. Laurikka, T. Lehtimaki, K. Leineweber, R. N. Lemaitre, M. Li, H. E. Lim, H. J. Lin, H. Lin, L. Lind, C. M. Lindgren, M. L. Lokki, B. London, R. J. F. Loos, S. K. Low, Y. Lu, L. P. Lyytikainen, P. W. Macfarlane, P. K. Magnusson, A. Mahajan, R. Malik, A. J. Mansur, G. M. Marcus, L. Margolin, K. B. Margulies, W. Marz, D. D. McManus, O. Melander, S. Mohanty, J. A. Montgomery, M. P. Morley, A. P. Morris, M. Muller-Nurasyid, A. Natale, S. Nazarian, B. Neumann, C. Newton-Cheh, M. N. Niemeijer, K. Nikus, P. Nilsson, R. Noordam, H. Oellers, M. S. Olesen, M. Orho-Melander, S. Padmanabhan, H. N. Pak, G. Pare, N. L. Pedersen, J. Pera, A. Pereira, D. Porteous, B. M. Psaty, S. L. Pulit, C. R. Pullinger, D. J. Rader, L. Refsgaard, M. Ribases, P. M. Ridker, M. Rienstra, L. Risch, D. M. Roden, J. Rosand, M. A. Rosenberg, N. Rost, J. I. Rotter, S. Saba, R. K. Sandhu, R. B. Schnabel, K. Schramm, H. Schunkert, C. Schurman, S. A. Scott, I. Seppala, C. Shaffer, S. Shah, A. A. Shalaby, J. Shim, M. B. Shoemaker, J. E. Siland, J. Sinisalo, M. F. Sinner, A. Slowik, A. V. Smith, B. H. Smith, J. G. Smith, J. D. Smith, N. L. Smith, E. Z. Soliman, N. Sotoodehnia, B. H. Stricker, A. Sun, H. Sun, J. H. Svendsen, T. Tanaka, K. Tanriverdi, K. D. Taylor, M. Teder-Laving, A. Teumer, S. Theriault, S. Trompet, N. R. Tucker, A. Tveit, A. G. Uitterlinden, P. Van Der Harst, I. C. Van Gelder, D. R. Van Wagoner, N. Verweij, E. Vlachopoulou, U. Volker, B. Wang, P. E. Weeke, B. Weijs, R. Weiss, S. Weiss, Q. S. Wells, K. L. Wiggins, J. A. Wong, D. Woo, B. B. Worrall, P. S. Yang, J. Yao, Z. T. Yoneda, T. Zeller, L. Zeng, S. A. Lubitz, K. L. Lunetta, and P. T. Ellinor. 2018. "Multi-ethnic genome-wide association study for atrial fibrillation." *Nat Genet* 50 (9):1225-1233. doi: 10.1038/s41588-018-0133-9.

Sakaue, S., M. Kanai, Y. Tanigawa, J. Karjalainen, M. Kurki, S. Koshiba, A. Narita, T. Konuma, K. Yamamoto, M. Akiyama, K. Ishigaki, A. Suzuki, K. Suzuki, W. Obara, K. Yamaji, K. Takahashi, S. Asai, Y. Takahashi, T. Suzuki, N. Shinozaki, H. Yamaguchi, S. Minami, S. Murayama, K. Yoshimori, S. Nagayama, D. Obata, M. Higashiyama, A. Masumoto, Y. Koretsune, FinnGen, K. Ito, C. Terao, T. Yamauchi, I. Komuro, T. Kadowaki, G. Tamiya, M. Yamamoto, Y. Nakamura, M. Kubo, Y. Murakami, K. Yamamoto, Y. Kamatani, A. Palotie, M. A. Rivas, M. J. Daly, K. Matsuda, and Y. Okada. 2021. "A cross-population atlas of genetic associations for 220 human phenotypes." *Nat Genet* 53 (10):1415-1424. doi: 10.1038/s41588-021-00931-x.

Smith, P. K., R. I. Krohn, G. T. Hermanson, A. K. Mallia, F. H. Gartner, M. D. Provenzano, E. K. Fujimoto, N. M. Goeke, B. J. Olson, and D. C. Klenk. 1985. "Measurement of protein using bicinchoninic acid." *Anal Biochem* 150 (1):76-85. doi: 10.1016/0003-2697(85)90442-7.

Stuart, T., A. Butler, P. Hoffman, C. Hafemeister, E. Papalexi, W. M. Mauck, 3rd, Y. Hao, M. Stoeckius, P. Smibert, and R. Satija. 2019. "Comprehensive Integration of Single-Cell Data." *Cell* 177 (7):1888-1902 e21. doi: 10.1016/j.cell.2019.05.031.

Weng, L. C., S. H. Choi, D. Klarin, J. G. Smith, P. R. Loh, M. Chaffin, C. Roselli, O. L. Hulme, K. L. Lunetta, J. Dupuis, E. J. Benjamin, C. Newton-Cheh, S. Kathiresan, P. T. Ellinor, and S. A. Lubitz. 2017. "Heritability of Atrial Fibrillation." *Circ Cardiovasc Genet* 10 (6). doi: 10.1161/CIRCGENETICS.117.001838.

Wolf, F. A., P. Angerer, and F. J. Theis. 2018. "SCANPY: large-scale single-cell gene expression data analysis." *Genome Biol* 19 (1):15. doi: 10.1186/s13059-017-1382-0.

Wolf, F. A., F. K. Hamey, M. Plass, J. Solana, J. S. Dahlin, B. Gottgens, N. Rajewsky, L. Simon, and F. J. Theis. 2019. "PAGA: graph abstraction reconciles clustering with trajectory inference through a topology preserving map of single cells." *Genome Biol* 20 (1):59. doi: 10.1186/s13059-019-1663-x.

Wolock, S. L., R. Lopez, and A. M. Klein. 2019. "Scrublet: Computational Identification of Cell Doublets in Single-Cell Transcriptomic Data." *Cell Syst* 8 (4):281-291 e9. doi: 10.1016/j.cels.2018.11.005.
