# Supplementary material for: Single-cell RNA sequencing reveals the contribution of smooth muscle cells and endothelial cells to fibrosis in human atrial tissue with atrial fibrillation
Source: Mol Med. 2024 Dec 19;30:247. doi: 10.1186/s10020-024-00999-1 (PMC11661033; doi:10.1186/s10020-024-00999-1)
Supplement: Supplementary file 8 — Supplementary Material 8 [file 10020_2024_999_MOESM8_ESM.docx]

**Supplementary Figures**


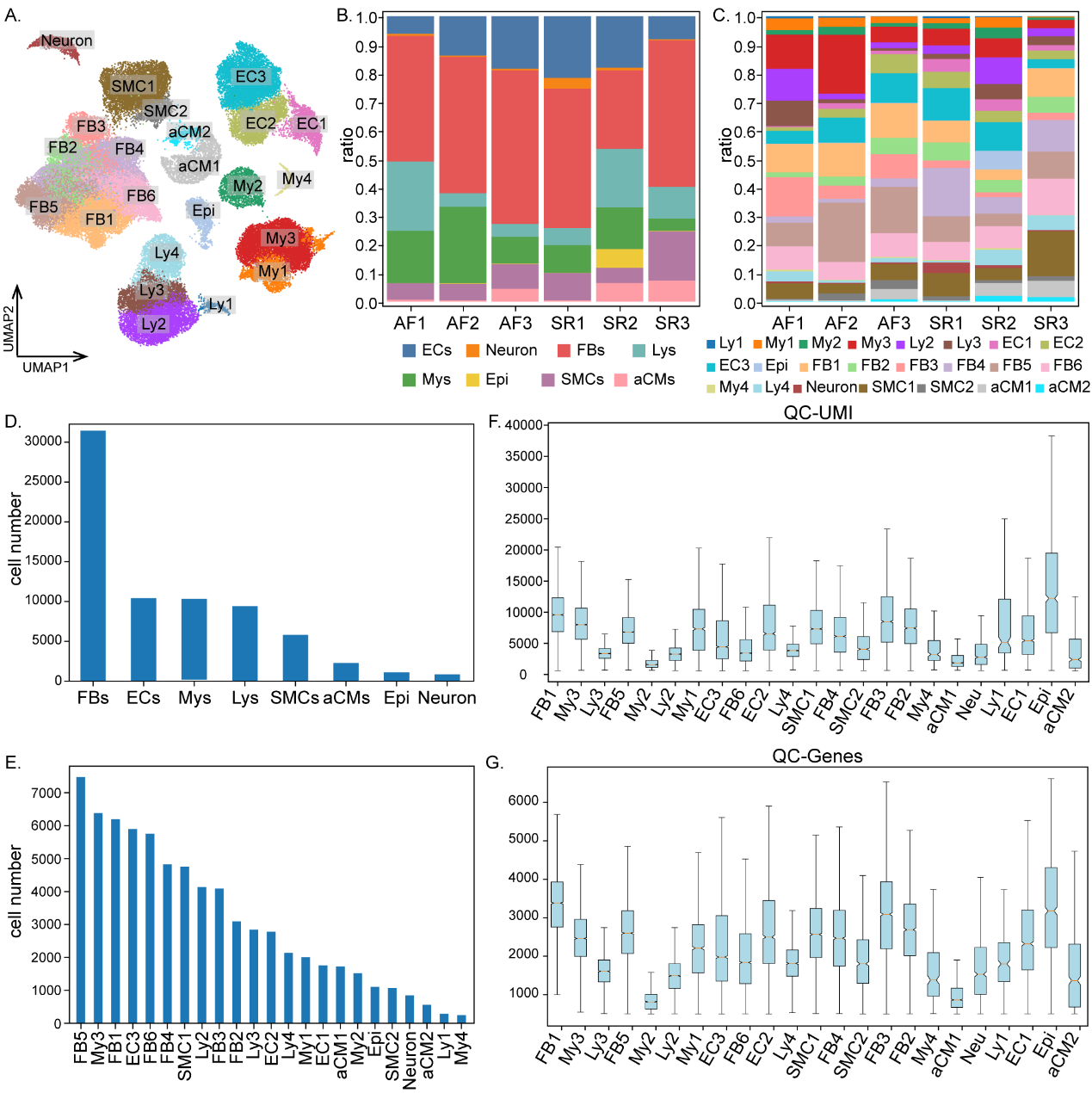


**Fig.s1 Refinement of population identities. A.** Unbiased clustering of 71,440 cells reveals 23 cellular subclusters. Subclusters are distinguished by different colors. **B.** Cell proportions of clusters in SR and AF. **C.** Cell proportions of subclusters in SR and AF. **D.** Distribution of number of cells per cluster. **E.** Distribution of number of cells per subcluster. **F.** Boxplots of QC-UMI in per subcluster. **G.** Boxplots of QC-genes expression per subcluster.

­­


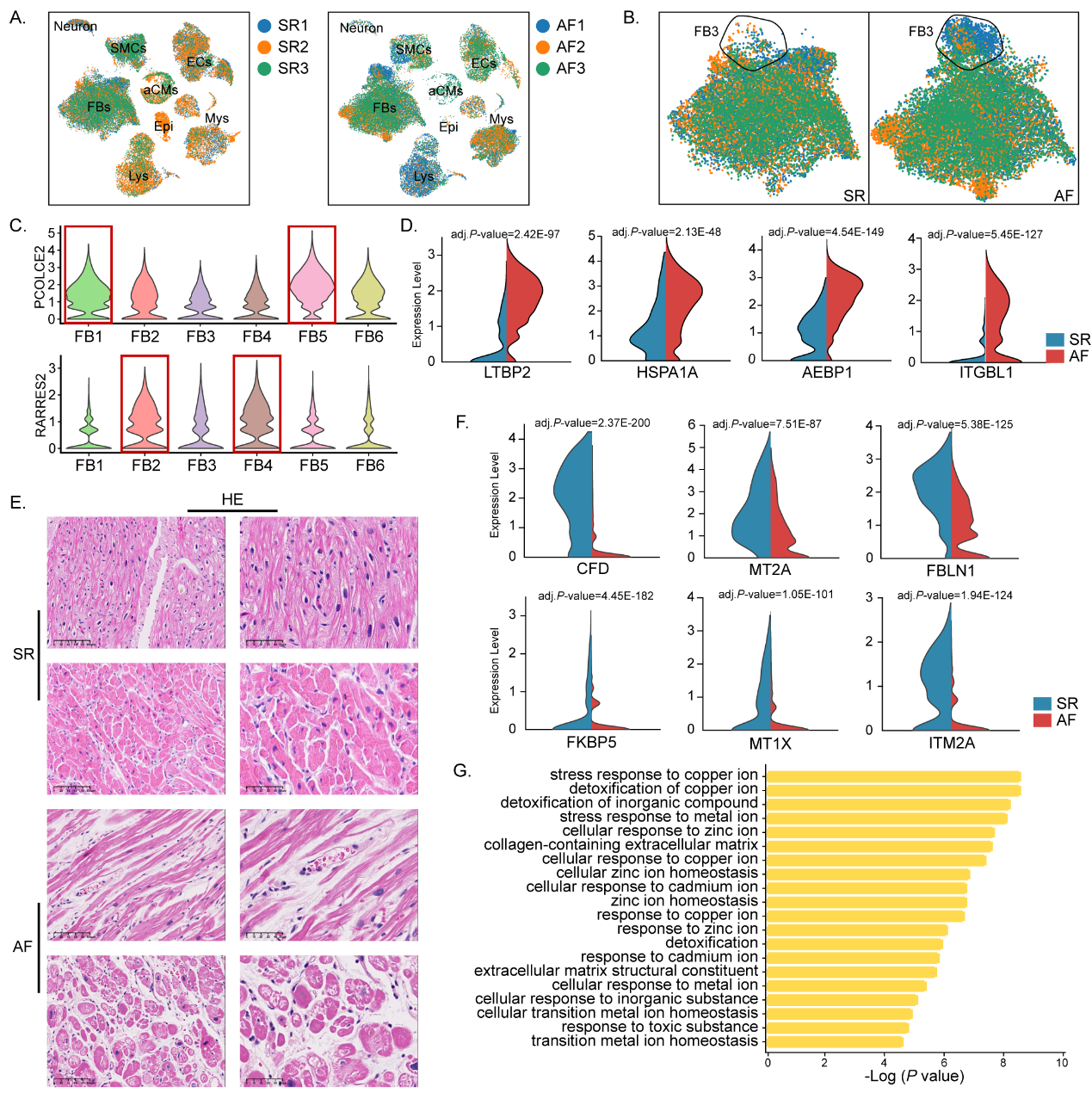


**Fig.s2 Fibroblasts. A.** Distribution of cell populations from SR (left) and AF (right). **B.** Distribution of SR and AF FB3 in the UMAP embeddings. **C.** PCOLCE2 and RARRES2 expression levels in cluster FBs. **D.** Violin plots showing representative differentially expressed genes between SR FB3 and AF FB3. **E.** Representative hematoxylin–eosin (H&E) staining in SR and AF (n = 3/group). **F, G.** Downregulated differentially expressed genes of AF in FB3 and GO Biological Process enrichment analysis.


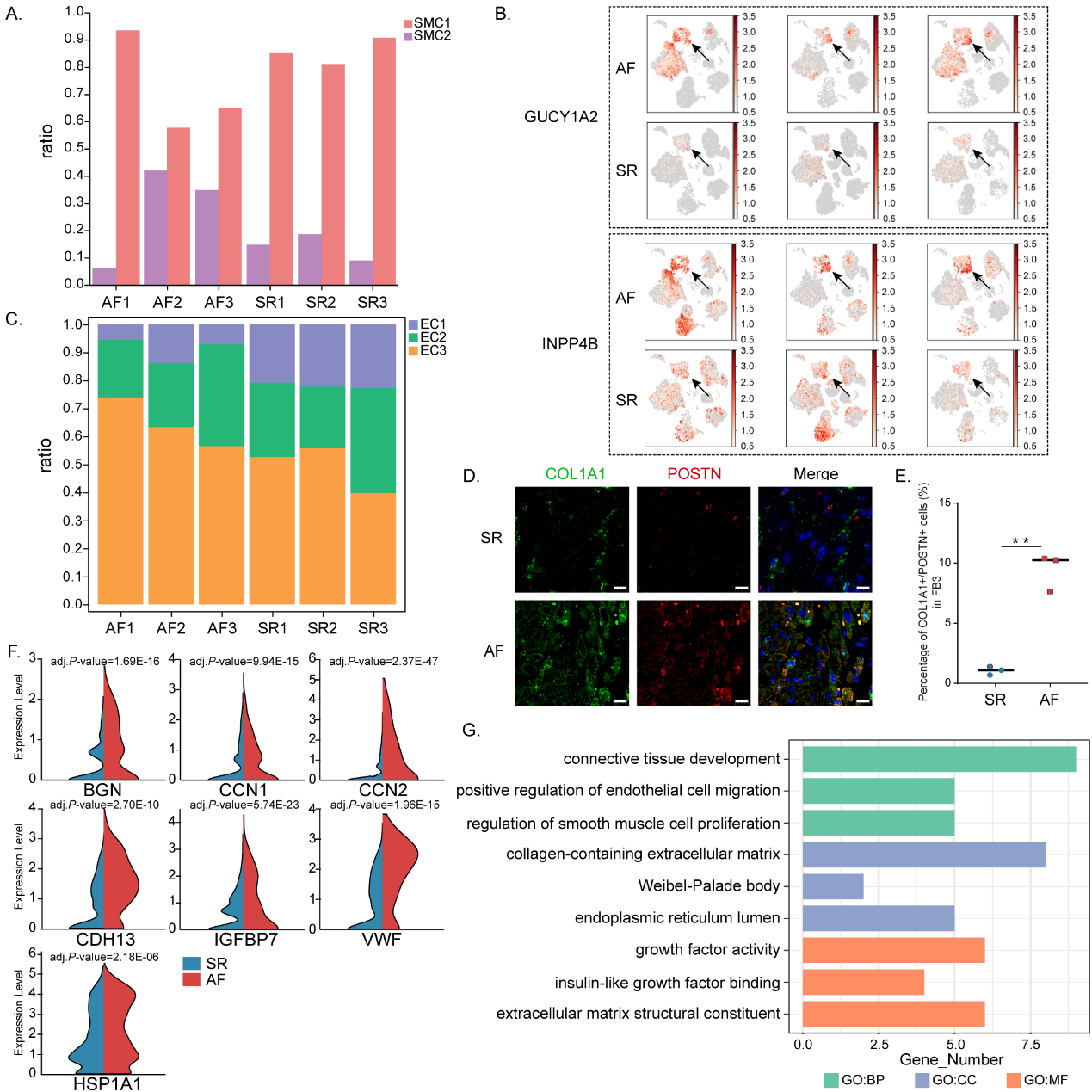


**Fig.s3 SMCs and ECs. A.** Cell proportions of SMCs in SR and AF. **B.** Feature plots of the expression distribution for GUCY1A2 and INPP4B in AF and SR. Expression levels for each cell are color-coded and overlaid onto the UMAP plot. **C.** Cell proportions of ECs in SR and AF. **D.** Fluorescence in Situ Hybridization of NPR3 and TXNDC5 in SR and AF. Scale bar = 20 μm. Percentage of NPR3+/TXNDC5+ cells in SR (n=3) and AF (n=3). Data are presented as mean values ± SD (n = 5 images examined over 3 independent experiments). Statistical analysis was performed using unpaired two-tailed Student’s t-test, ***P* = 0.0008. **F.** Violin plots showing representative upregulated differentially expressed genes in AF EC1. **G.** GO enrichment terms of upregulated differentially expressed genes in EC1.


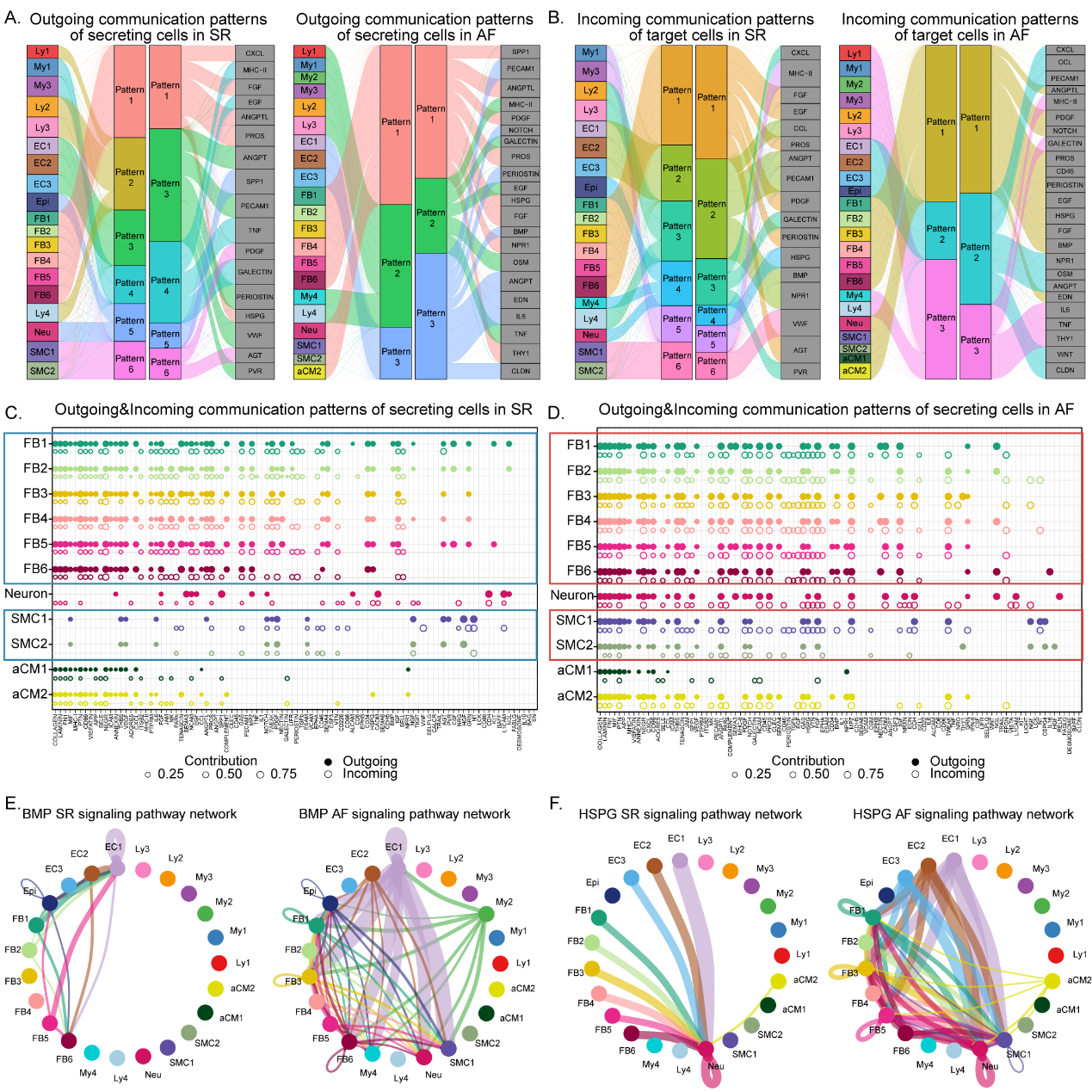


**Fig.s4 Application of CellChat to communications between cell populations. A.** The outgoing signaling patterns of secreting cells in SR and AF visualized by alluvial plot, which shows the correspondence between the inferred latent patterns and cell groups, as well as signaling pathways. The thickness of the flow indicates the contribution of the cell group or signaling pathway to each latent pattern. The height of each pattern is proportional to the number of its associated cell groups or signaling pathways. Outgoing patterns reveal how the sender cells coordinate with each other, as well as how they coordinate with certain signaling pathways to drive communication. **B.** Incoming signaling patterns of target cells. Incoming patterns show how the target cells coordinate with each other, as well as how they coordinate with certain signaling pathways to respond to incoming signaling. **C, D.** The dot plot showing the comparison of outgoing & incoming signaling patterns of secreting cells between SR and AF. The dot size is proportional to the contribution score computed from pattern recognition analysis. Higher contribution score implies the signaling pathway is more enriched in the corresponding cell group. **E, F.** BMP and HSPG signaling pathway network in SR and AF.


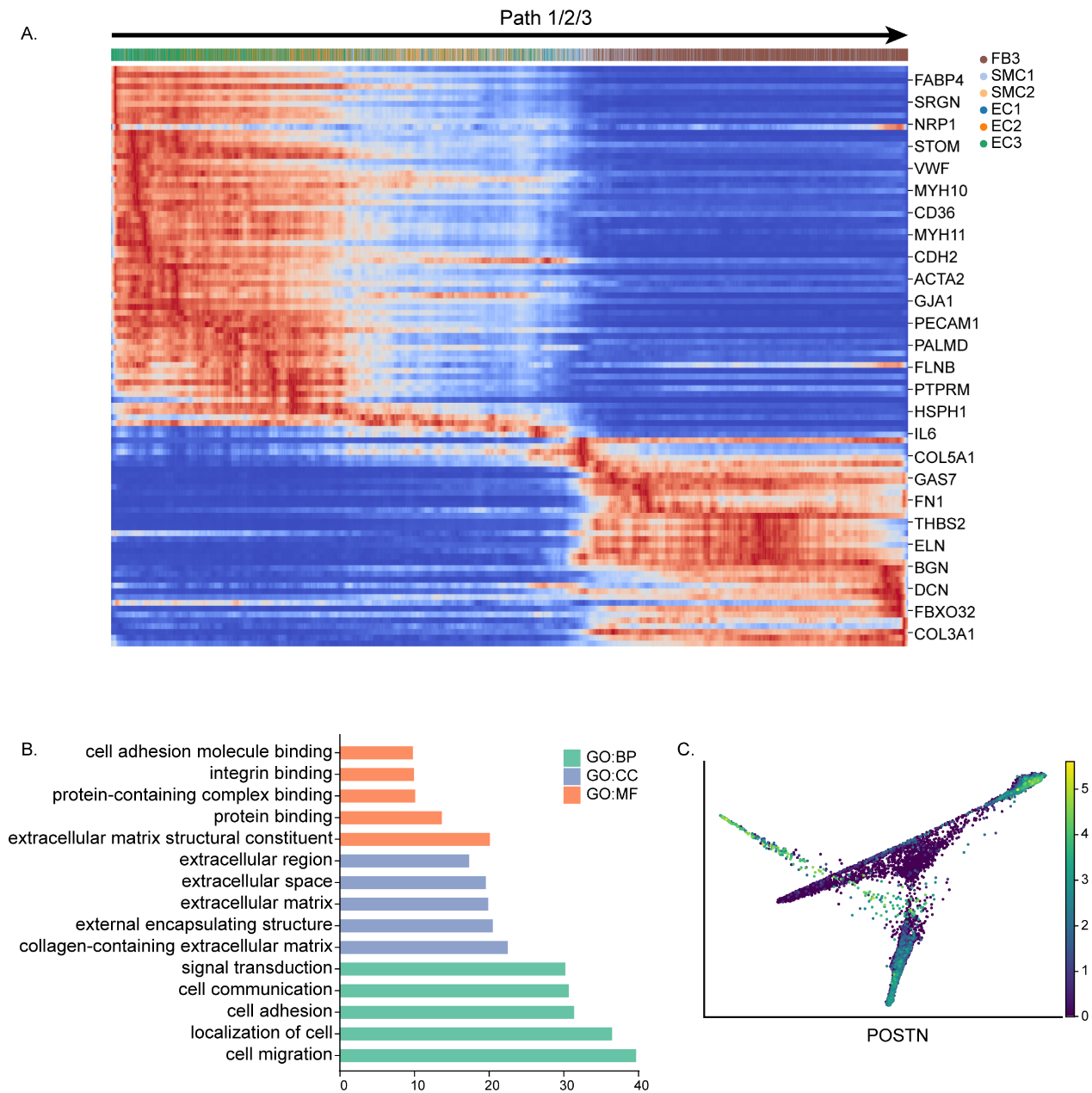


**Fig.s5 PAGA predicts developmental trajectories. A.** Heatmaps of pseudotime-dependent gene expression. **B.** Selected top GO terms related to corresponding DEGs in A. **C.** Feature plots of expression distribution for POSTN across pseudotime. Expression levels for each cell are color-coded.


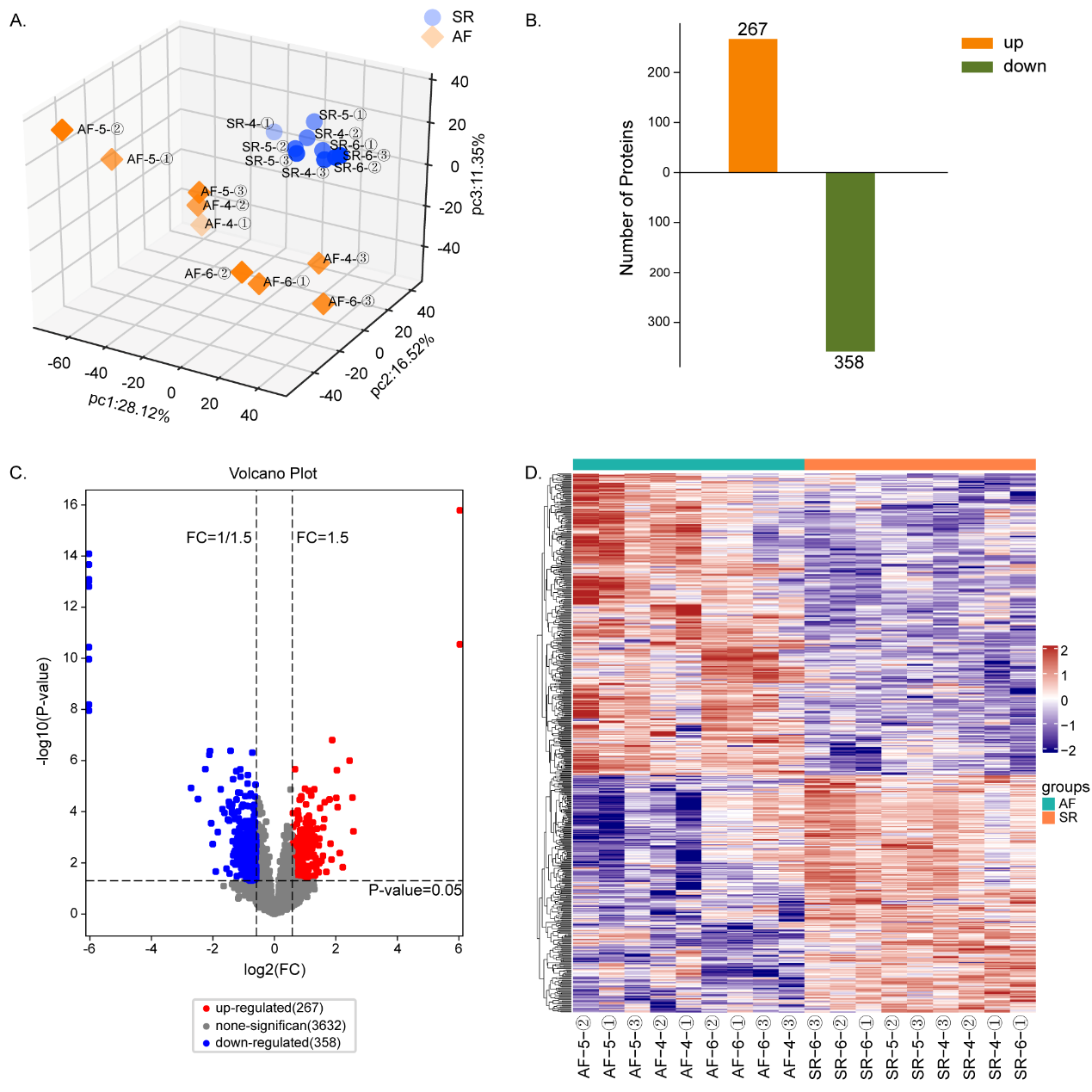


**Fig.s6 Proteomic data quality control and differential protein expression. A.** Principal component analysis (PCA) of trusted protein expression among different samples. Three samples were tested for each patient. **B.** Bar chart of differentially expressed proteins for the overall distribution of differentially expressed proteins. **C.** Differential protein expression was verified using Student's t-test and a volcano plot was constructed. Blue dots indicate down-regulated differentially expressed proteins, red dots indicate up-regulated differentially expressed proteins, and gray dots indicate non-significantly differentially expressed proteins. **D.** Clustering heat map obtained by clustering according to protein expression level. Red represents high expression protein, blue represents low expression protein, and each row represents the expression level of each protein in different groups.
